# Gene expression profiling of aging reveals activation of a p53-mediated transcriptional program

**DOI:** 10.1186/1471-2164-8-80

**Published:** 2007-03-23

**Authors:** Michael G Edwards, Rozalyn M Anderson, Ming Yuan, Christina M Kendziorski, Richard Weindruch, Tomas A Prolla

**Affiliations:** 1 Department of Genetics and Medical Genetics, University of Wisconsin, Madison, WI, USA; 2 Veterans Administration Hospital, University of Wisconsin, Madison, WI, USA; 3 School of Industrial and Systems Engineering, Georgia Institute of Technology, Atlanta, GA, USA; 4 Department of Biostatistics and Medical Informatics, University of Wisconsin, Madison, WI, USA; 5 Department of Medicine and Wisconsin Primate Research Center, University of Wisconsin, Madison, WI, USA

## Abstract

**Background:**

Aging has been associated with widespread changes at the gene expression level in multiple mammalian tissues. We have used high density oligonucleotide arrays and novel statistical methods to identify specific transcriptional classes that may uncover biological processes that play a central role in mammalian aging.

**Results:**

We identified 712 transcripts that are differentially expressed in young (5 month old) and old (25-month old) mouse skeletal muscle. Caloric restriction (CR) completely or partially reversed 87% of the changes in expression. Examination of individual genes revealed a transcriptional profile indicative of increased p53 activity in the older muscle. To determine whether the increase in p53 activity is associated with transcriptional activation of apoptotic targets, we performed RT-PCR on four well known mediators of p53-induced apoptosis: *puma*, *noxa*, *tnfrsf10b *and *bok*. Expression levels for these proapoptotic genes increased significantly with age (P < 0.05), while CR significantly lowered expression levels for these genes as compared to control fed old mice (P < 0.05). Age-related induction of p53-related genes was observed in multiple tissues, but was not observed in young SOD2^+/- ^and GPX4^+/- ^mice, suggesting that oxidative stress does not induce the expression of these genes. Western blot analysis confirmed that protein levels for both p21 and GADD45a, two established transcriptional targets of p53, were higher in the older muscle tissue.

**Conclusion:**

These observations support a role for p53-mediated transcriptional program in mammalian aging and suggest that mechanisms other than reactive oxygen species are involved in the age-related transcriptional activation of p53 targets.

## Background

Aging in skeletal muscle is characterized by loss of motor neurons, variations in size and type of muscle fibers, infiltration of fat and connective tissue, and a overall decrease in muscle mass (sarcopenia) [1]. Sarcopenia, the leading cause of frailty and disability in the elderly, has also been linked to other age-associated pathology, such as osteoporosis and impaired thermoregulation [2-5]. Several biological processes, such as an increase in inflammatory cytokines and a decrease in sex and growth hormones with age [6-8], have been suggested as contributing factors to the aging of skeletal muscle. At the cellular level, older muscle is associated with decreased protein synthesis [9], an increase in DNA, protein and lipid oxidation [10,11], and the accumulation of mitochondrial abnormalities [12].

Several studies have employed microarray technology to uncover changes in gene expression that accompany aging in skeletal muscle of mice [13], rats [14], monkeys [15] and humans [16,17]. When taken as a whole, results obtained from these studies suggest that the expression of genes indicative of cellular damage, such as those involved in the stress or inflammatory response, increase with age, while expression of metabolic and biosynthetic genes decrease with aging. We had previously investigated age-related changes in gene expression of mouse gastrocnemius tissue using high density oligonucleotide arrays [13], but our study was limited by low sample number (3 mice per age group), an arbitrary fold change criteria (> 1.5 fold difference) with no statistical analysis to determine differentially expressed genes, and a limited survey of genes expressed in the mouse genome (6347 transcripts). We therefore decided to expand our initial investigation using a larger sample size (5 mice per age group), a stringent statistical criteria to determine differentially expressed genes [posterior probability (PP) > 0.90], and a greater representation of the mouse genome (22,690 transcripts). Our current microarray study of young and old C57BL/6NHsd mice supports previous findings on specific transcript classes differentially expressed with aging and also suggests that increased p53 activity in skeletal muscle of older animals may contribute to aging phenotypes. Caloric restriction, which extends the life span of this strain of mice by approximately 40%, prevents the majority of the changes in gene expression observed in the older mice under the control diet.

## Results

### General

We used an empirical Bayes (EB) hierarchical modeling approach called EBarrays to identify 712 genes differentially expressed [posterior probability (PP) > .90] in old mouse gastrocnemius tissue [see additional file 1]. There are a number of advantages to our statistical approach. The main ones are the ability to combine information across genes and the dual nature of posterior probabilities. The posterior probabilities quantify evidence in favor of differential expression; however, unlike classic p-values, they can also be used to measure evidence in favor of equivalent expression. Measurements taken from gastrocnemius tissue from a group of old mice on caloric restriction (CR, n = 5), a regimen previously shown to increase lifespan and retard aging parameters in mice, was used as a negative control to aging. There were 311 genes with higher expression in the young animals and 401 genes with greater expression in the old. As expected, CR completely or partially reversed 87% of the genes with age-associated changes in expression (partial CR reversal was determined as > 25% CR effect). In agreement with previous microarray studies done on skeletal muscle of mice, monkeys and humans, we also found that genes associated with metabolic functions, particularly energy pathways, were higher in young control animals (Table 1). In contrast, genes indicative of cellular damage, such as stress response and apoptotic genes, had higher expression levels in the muscle tissue of older animals (Table 1).

Among the biological classes of transcripts with an age effect, there were several gene families whose mRNA levels were significantly increased or decreased with age [see additional file 1]. There were 8 procollagen genes (11 probe sets) that were altered with aging; 7 of these showed higher levels in the young muscle (*col1a1, col1a2, col3a1, col5a1, col6a1, col6a2, col6a3*) while only one procollagen gene was increased with aging (*col17a1*). The keratins, important cytoskeletal structural proteins, were represented by 8 genes (9 probe sets) all of which displayed increased transcript levels in older muscle. Three members (alpha, beta and delta) of the CCAAT/enhancer-binding protein (CEBP) family of transcription factors had increased levels of transcripts in the older mice. The encoded CEBP alpha, beta and delta proteins are important in the regulation of genes involved in immune and inflammatory responses, cell cycle arrest and body weight homeostasis (for a review, see [18]). Because many genes are represented more than once on the MOE430A Affymetrix microarray, several genes had multiple hits on our list of genes with an age effect in skeletal muscle. Among those genes with four or more independent listings were: *fibromodulin *(6 probe sets, all with reduced expression in the old), *transferrin receptor *(5 probe sets, all with reduced expression in the old), *immunoglobulin kappa chain variable 8 *(4 probe sets, all with increased expression in the old), and *myristoylated alanine rich protein kinase C substrate *(*marcks*) (4 probe sets, all with reduced expression in the old).

### Transcript and Protein Levels of p53-Related Genes Altered with Aging

Given that skeletal muscle is largely a post-mitotic tissue, we were surprised to find that a large number of genes involved in the regulation of the cell cycle had increased mRNA levels with age (5.2% of all genes with a statistically significant effect). Two gene products that are known to play a critical role in initiating cell cycle arrest following DNA damage, cyclin-dependent kinase inhibitor 1A (p21) and growth arrest and DNA-damage-inducible, alpha (GADD45a), were both increased significantly in the skeletal muscle of the aged mice. Expression levels for *p21 *and *GADD45a *were 1.9 and 1.8 fold higher respectively in older mouse compared to the younger animals. An age-related increase in *p21 *has previously been observed in muscle of both male and female humans (2.9 and 4.0 fold respectively) [16,17] and monkeys (4.1 fold) [15], while the expression of *GADD45a *has been shown to be elevated in mouse gastrocnemius muscle (2.6 fold) [13] and both male and female human muscle (2.0 and 1.9 fold respectively) [16]. Because the expression of these two genes is typically mediated by the action of p53, we decided to systematically examine our list of genes differentially expressed between young and old to determine if there were any other transcripts that are also connected to p53 activity. As shown in Table 2, the transcripts that are activated by p53, or whose gene products are known to bind to p53, increase significantly with age, while the expression of genes that are known to inhibit p53 activity decline in old muscle. The average CR prevention on these p53-related genes was 77%.

**Figure 1:**
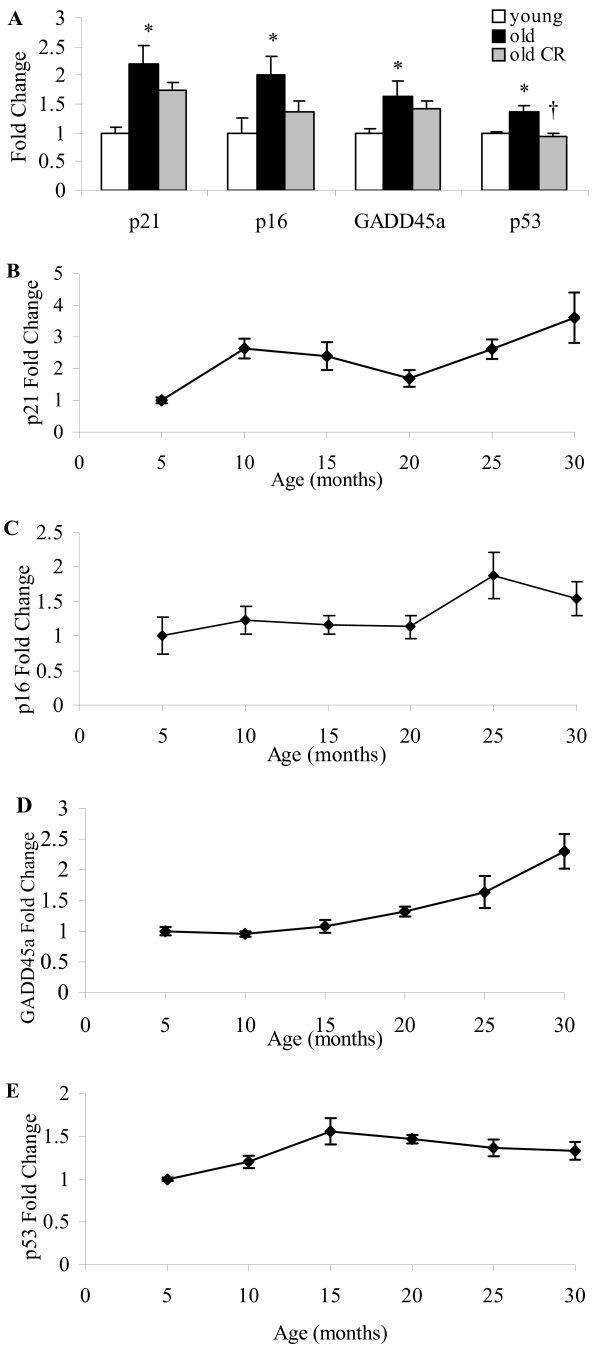
***p21*, *p16*, *Gadd45a *and *p53 *expression in mouse skeletal muscle**. (A) Relative expression ratio ± SE for mRNA levels, as determined by RT-PCR, for *p21*, *p16*, *Gadd45a *and *p53 *in young (5 month old), old (25 month old), and old CR (n = 5 for each group). (B-E) Relative expression ratio ± SE for mRNA levels for these genes over the course of the adult mouse life span (5 to 30 months of age). The mean of 5 mice ± SE was used for each data point on the graphs. Two-tailed Student's *t*-test; *P < 0.05 (young versus old) and ^†^P < 0.05 (old verses old CR).

To confirm the age-related changes in expression observed in *p21 *and *GADD45a *using microarrays, we probed young, old and old CR mice using RT-PCR (Figure 1A). We also examined the expression levels of the *p53 *gene itself, along with the expression levels of another important tumor suppressor, *p16*. Both *p53 *and *p16 *transcripts were considered absent (below levels of background hybridization) in all animals according to the Affymetrix software, but we were able to detect expression using the more sensitive method of RT-PCR. As is shown in Figure 1A, transcript levels for these genes increased significantly (Student's *t*-test, P < 0.05) with age. Furthermore, old animals undergoing CR demonstrated reduced levels for these genes compared to age-matched mice fed normal diets (only the difference in *p53 *levels was considered significant at P < 0.05). To examine how the expression of *p21*, *p16*, *GADD45a *and *p53 *is altered over the course of the adult mouse life span, we used RT-PCR to measure transcript levels for these genes in gastrocnemius muscle at 5 month intervals from 5 to 30 months of age (Figure 1B–E). With the exception of *GADD45a *(Figure 1D), the increase in expression of these genes does not show a linear pattern with age. *p16 *and *GADD45a *expression increases significantly only in the last third of the lifespan, whereas expression of *p53 *and its transcriptional target *p21 *occur as early as 10 months of age.

**Figure 2:**
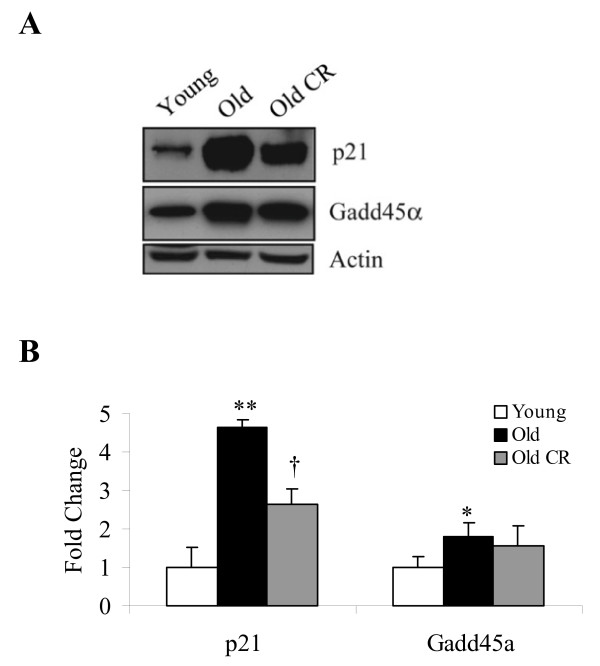
**Protein levels for p21 and GADD45a in mouse skeletal muscle**. (A) Representative western blot for p21 and GADD45a staining in young, old and old CR skeletal muscle. (B) Expression ratio information on protein levels (n = 3 and n = 4 for p21 and Gadd45a respectively for each age and diet). One-tailed Student's *t*-test; *P < 0.05, **P < 0.005 (young verses old), ^†^P < 0.005 (old versus old CR).

To determine whether the p53-mediated transcriptional program observed in older mouse skeletal muscle translated into higher levels of p53-associated proteins being expressed, we probed young, old and old CR muscle for p53-transcriptional targets p21 and GADD45a. Western immunoblot confirmed a significant increase in p21 protein (4.6 fold, P < 0.005) and GADD45a (1.6 fold, P < 0.05) in older mouse skeletal muscle (Figure 2). Levels for these proteins were reduced in the old CR mice as compared to old mice on the control diet (approximately 57% and 77% of old levels for p21 and GADD45a respectively). An attempt was made with several antibodies to measure p53 protein levels (both phosphorylated and non-phosphorylated isoforms) in GN muscle, but the signal from the Western blots was either absent or too low to accurately quantitate p53 in any age group (data not shown).

### Transcript Levels of *p21*, *p16 *and *GADD45a *in Mouse Models of Increased Oxidative Stress

To test whether the changes in expression of the p53-related genes could be attributed to cumulative oxidative damage with age, we compared transcript levels for *p21*, *p16*, *GADD45a *and *p53 *in C57BL/6 mice with a heterozygous knockout for either glutathione peroxidase 4 (*GPX4*^+/-^) (5 mo old) or MnSOD (*SOD2*^+/-^) (~9 mo old) mutations to age-matched wild-type mice. *GPX4*^+/-^mice have approximately half the amount of mRNA and protein levels for this essential Se-dependent antioxidant protein as compared to wild type mice [19]. Mouse embryonic fibroblasts (MEFs) derived from mice heterozygous for the *gpx4 *gene show increased evidence of lipid and DNA oxidation, and are sensitive to gamma irradiation [19,20]. *SOD2*^+/- ^mice show a ~50% reduction in MnSOD protein content and MnSOD activity in gatrocnemius tissue [21] and have increased oxidative damage as demonstrated by significantly elevated levels of 8-oxo-2-deoxyguanosine (8oxodG) in both nuclear and mitochondrial DNA in all surveyed tissues compared to wild type mice [22]. RT-PCR analysis of *p21*, *p16*, *GADD45a *and *p53*, in *GPX4*^+/- ^mice showed a significant increase (P < 0.005) in p53 expression compared to the age-matched wild-type mice. We did observe a ~2 fold increase in p16, although it was not statistically significant (P < 0.12). Transcript levels of these genes were not significantly different between *SOD2*^+/- ^mice and age-matched controls [see additional file 2]. Thus, constitutive oxidative damage does not always trigger the expression of the genes examined. Possibly, different types of DNA damage in GPX4-/+ and SOD2+/- mice explain the observed differences.

### Increased p53 Levels in Older Muscle is Associated with Increased Expression of Genes Involved in p53-Mediated Apoptosis

**Figure 3:**
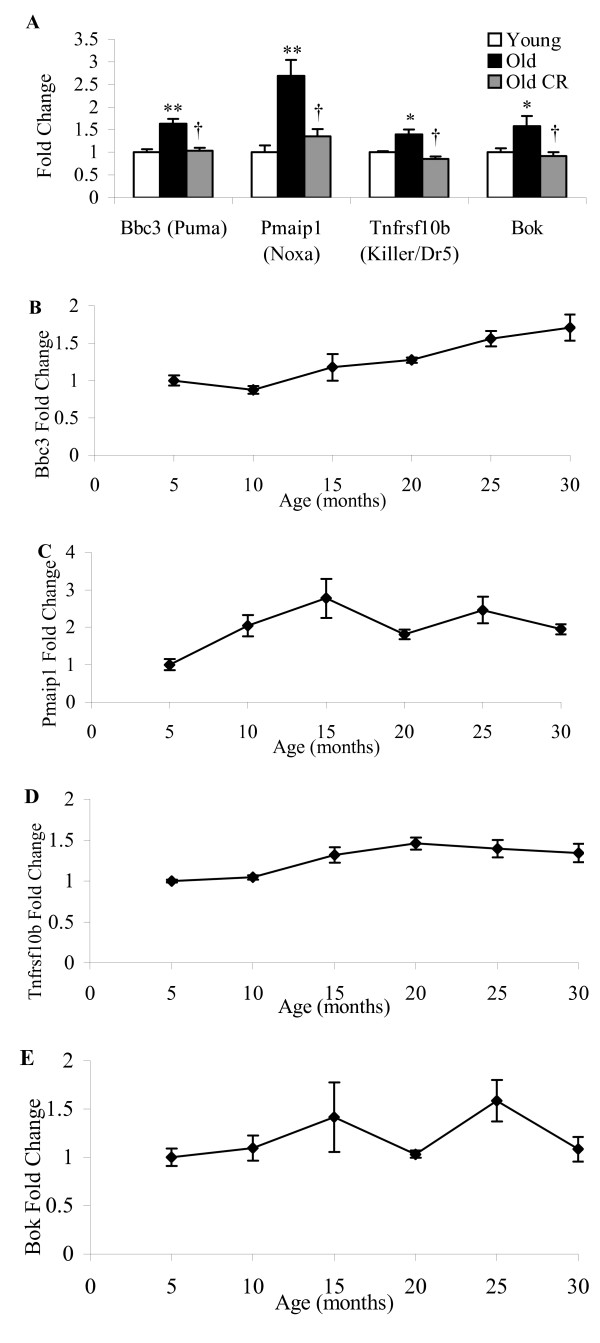
**RT-PCR of proapoptotic *bbc3*, *pmaip1*, *tnfrsf10b *and *bok *expression in mouse skeletal muscle**. (A) Relative expression ratio ± SE for mRNA levels of p53-mediated apoptotic genes in young, old, and old CR (n = 5 for each group). The mRNA levels for these four genes were also measured using RT-PCR in 5–30 month old C57BL/6NHsd mouse gastrocnemius muscle (B-E) (mean ± SE of 5 animals per age group). Two-tailed Student's *t*-test; *P < 0.05, **P < 0.005 (young verses old), ^†^P < 0.05 (old versus old CR).

A critical function of p53 is the induction of apoptosis in cells that have undergone DNA damage or exhibit uncontrolled cellular growth [23]. To investigate whether increased p53 activity with age is associated with transcription profiles indicative of apoptosis in old gastrocnemius muscle, we examined mRNA levels of four genes known to be key transducers of p53-mediated apoptosis: *Bcl-2 binding component 3 *(*bbc3/puma*) [24], *phorbol-12-myristate-13-acetate-induced protein 1 *(*pmaip1/noxa*) [25], *tumor necrosis factor receptor superfamily, member 10b *(*tnfrsf10b/killer/dr5*) [26] and *Bcl-2-related ovarian killer protein *(*bok*) [27]. We observed a significant increase in mRNA levels with age for these genes in mouse gastrocnemius muscle and a significant decrease in levels due to caloric restriction in old, age-matched controls (Figure 3A). The expression levels for all of these p53-mediators of apoptosis were also examined in the gastrocnemius muscle over the course of the mouse adult lifespan (Figure 3B–E), and increased levels of transcript were usually observed in the first half of the lifespan. We also compared the levels for these proapoptotic genes in the gastrocnemius tissue of *GPX4+/- *and the *SOD+/- *mice to age-matched wild type mice using RT-PCR. Only the levels for *tnfrsf10b *were considered significantly higher (P < 0.005) in the *GPX4+/- *mice while expression of these genes was not significantly different in the *SOD2+/- *mice [see additional file 3]. These findings indicate that p53-dependent mediators of apoptosis are regulated with age and that the mechanism of activation is independent of endogenous levels of oxidative stress.

### Increase in mRNA Levels of p53 and p53-Associated Genes in Multiple Tissues of Old C57Bl6/J Mice

To determine whether the p53-mediated transcriptional program we observe in older skeletal muscle may also occur in other tissues, we used RT-PCR to measure mRNA levels of p53-associated genes in heart, lung, liver, kidney, brain and intestine of young, old, and old CR mice (Table 3). Transcript levels for *p21*, *p16*, *p53 *and *pmaip1 *demonstrated increased levels in multiple tissue types in the old mice, while an increase in transcript levels for *gadd45a*, *bbc3*, *tnfrsf10b *and *bok *was only observed in old skeletal muscle. These findings suggest that activation of a p53-mediated transcriptional program may be a common feature of aging in multiple tissues, but the pattern of expression of p53-dependent genes is tissue specific.

## Discussion and conclusion

Our initial investigation of muscle aging in mice revealed many differences in the transcriptional patterns between young and old mice [13]. In general, transcript levels for genes that are associated with cellular damage were elevated in older muscle, while transcript levels for genes involved in energy metabolism were reduced with age. The current study further demonstrates that the intrinsic transcriptional patterns are altered with aging in mouse skeletal muscle. As with other microarray studies done on older mammalian muscle [13-17], we have also found a disproportionate number of metabolic genes, such as those involved in lipid and carbohydrate metabolism, decrease in transcription levels with age. The age-related decrease in expression of these energy-associated classes likely reflects the reduction in mitochondria observed in older gastrocnemius muscle. For example, a 23–25% reduction in mtDNA copy number and a corresponding 17–22% reduction in the expression of mitochondrial DNA-encoded cytochrome c oxidase (COX) subunits I and III was previously reported in old rat gastrocnemius tissue [28]. In our study, out of 25 transcripts that are associated with the mitochondria, the expression of 18 of these probe sets are significantly reduced with age (PP < .90) [see additional file 1]. Conversely, genes associated with the stress and immune response, such as those involved in the induction of apoptosis or the response to DNA damage, have elevated mRNA levels in the older animals. The age-associated increase in genes that are activated during cellular stress and injury is probably in response to a wide variety of biological damage known to accumulate in older muscle over the course of the lifespan [9,10,12]. We also observed that the expressions of a large number of genes involved in the regulation of the cell cycle were increased with age in mouse skeletal muscle. A closer examination of these cell cycle genes revealed a pattern of expression indicative of increased p53 activity in older mouse muscle (Table 2).

Welle et al. also found transcriptional evidence of increased p53 activity with age in the skeletal muscle of young (20–29 yrs. old) and old (65–71 yrs. old) women [16]. In their study, genes that regulate or enhance p53 activity (*Pin1, p57 *and *Wrn*) or that are induced by p53 (*p21 *and *GADD45a*) demonstrate increased expression in older female muscle. Based on this evidence, Welle and coworkers suggested that future studies should examine the possibility that p53 levels or activity are increased in older muscle. One possible mechanism for the observed increase in p53 activity is elevated levels of DNA damage with age. The concentration of 8-oxo-2 deoxyguanosine (oxo8dG), a byproduct of DNA oxidation, has been observed to increase with age in a variety of tissues, including skeletal muscle, in rats and various strains of mice (CR reduced the levels of oxo8dG in most of these tissues) [29]. We tested this hypothesis by examining the levels of p53 mRNA and related transcripts in transgenic mice with half the wild type levels of antioxidants GPX4 or SOD2. These mice have elevated levels of oxidative DNA damage yet failed to show any significant increase in expression of *p21 *and *GADD45a*, two well studied p53-induced genes [see additional file 2]. The *GPX4*^+/- ^mice did display a significant increase in transcript levels for p53 as compared to age-matched controls, but no change was observed for the *SOD2*^+/- ^mice. Possibly, different types of DNA damage impact the expression of these genes differently. Another possibility is that the DNA damage signaling that is the trigger for activation of p53 in old muscle originates from chromosomal damage, such as double-strand breaks or telomere shortening, rather than from oxidative alterations. We also note that there are two likely possibilities for the cellular origin of the alteration in expression of cell cycle regulators. One is that these alterations in gene expression reflect a decline in proliferation of a muscle-associated cell such as the satellite cell. It has recently been reported that hematopoietic stem cell numbers are higher in old mice with reduced p53 activity (p53+/-) and fail to increase with age in old mice with hypermorphic p53 activity (p53+/m) as compared to their wild type counterparts [30]. A second possibility is that the expression of these genes reflects a general p53-mediated DNA damage response associated with age-related DNA modifications in myocytes.

Many recent studies have linked tumor suppressor activity, in particular the tumor suppressor p53, to organismal aging [23,31-34]. p16^INK4a^, a product of the INK4a/ARF (Cdkn2a) locus also increases in expression with age in most tissues as shown in previous studies [35-37] and also in the current analysis. This increase in p16^INK4a ^expression with age has recently been shown to contribute to age-dependent decline in regenerative potential of pancreatic islet regenerative capacity [38], decreases in forebrain progenitors and neurogenesis [39] and the decrease in hematopoietic stem cell repopulation defects [40]. However, we note that it is possible that only rare cells in tissues express p16^INK4a ^with aging. Under these conditions, a large effect of increased ROS in a specific biologically relevant muscle compartment (e.g. satellite cells) could be missed by studying unfractionated muscle as we did in our analysis. Thus, the induction of a cell senescence and apoptotic program with age may underlie key features of the aging process.

Given our finding that mRNA levels for p53-associated genes were elevated in multiple tissues of old mice (Table 3), our data suggests that increased p53 activity is not limited to aging muscle but is also an overall consequence of organismal aging. Increased p53 mRNA levels is observed in the grey and white matter of the cortex, cerebellum, and medulla oblongata of old (24 month old) verses young (3.0–3.5 months old) rat brains and an increase in p53 protein levels is observed in the grey matter of old rats [41]. We did not find a significant difference in p53 gene expression between young and old brains, but our analysis was performed on whole brain homogenates and therefore any age-effect on p53 expression within the different regions of the brain might have been missed. Further support for the role of increased p53 activity contributing to aging process comes from transgenic studies in mice that express overactive p53 and also demonstrate accelerated aging [42,43]. Mice with augmented p53 function develop typical age-associated phenotypes such as delayed wound healing, reduced hair growth and reduced subcutaneous adipose tissue much earlier than their p53 wild type counterparts. Conversely, the long-lived p66Shc^-/- ^mouse, shown to have a 30% extended life span, exhibits impaired p53 apoptotic signaling [44]. Our study demonstrates that the majority of the p53-related transcriptional changes observed in old skeletal muscle are either completely or partially reversed through caloric restriction, a proven method to extended longevity. Based on the evidence for the involvement of the p53 pathway in organismal aging, it follows that p53 and p53 transcriptional targets have the potential to be good biomarkers of aging in multiple tissue types.

It has been well documented that there is a significant decrease in muscle mass with age, yet little is known as to the actual mechanism for this loss of tissue. It has been hypothesized that the decrease in muscle fiber can be attributed to increased apoptosis with age. In fact, an increase in biological markers of apoptosis, such as DNA fragmentation and proapoptotic caspase-type proteins, are increased with age in human [45] and rat skeletal muscle [46,47]. However, the mechanism for this age-associated apoptosis is unknown. It has been suggested that increased oxidative damage to the mitochondria, along with sarcoplasmic reticulum stress in older skeletal muscle could initiate caspase activation and lead to the apoptotic pathway that ultimately results in the destruction of the aged myocyte [47]. Moreover, Dirks and Leeuwenburgh also observed a significant reduction in DNA fragmentation (mono- and oligonucleosomes) and caspase levels in old (26 month old) rats on lifelong caloric restriction when compared to their age-matched, ad libitum-fed counterparts. The authors suggest the reduction of apoptosis in response to caloric restriction is due to reduced mitochondrial oxidant production and nuclear oxidative DNA damage in skeletal muscle [48]. Our results do not support this hypothesis. Although young *GPX4+/- *mice did show increased levels of expression, compared to wild-type young mice, of p53-associated mediator of apoptosis *tnfrsf10b*, little or no change was observed for *bbc3 *and *bok *(only *tnfrsf10b *was considered significant at P < 0.05). The *SOD2+/- *mice, on the other hand, demonstrated almost identical levels of expression for all of the surveyed p53 mediators of apoptosis to the age-matched, wild type controls. It is possible that increased oxidative damage may play a role in p53-mediated apoptosis in older muscle, but our data suggests that it may not be the sole causative effect. Old calorie restricted mice displayed a significant decrease in expression levels of all four of the p53-mediators of apoptosis compared to the normally fed old mice. It is clear that caloric restriction has an inhibitory effect on age-associated apoptosis of skeletal muscle, but this prevention may not be completely explained by the reduction of endogenous reactive oxygen species and the resulting decrease in oxidative DNA damage in these animals.

The role of p53 in the aging process of skeletal muscle is likely to be a complex process involving many different cellular effectors. Various proteins and pathways interact with p53 to modulate its activity. Furthermore, there are numerous p53-transcriptional targets, each with their own associated modulators that may influence muscle aging. As evidence of this complexity, we show that the expression profiles for p53-regulated genes are not identical to that of p53, as well as to each other, over the course of the mouse adult lifespan in gastrocnemius muscle (Figure 1B–E, Figure 3B–E). Future studies will concentrate on determining which specific components of the p53 pathway play a functional role in muscle aging, and organismal aging in general.

## Methods

### Animal treatments

Male C57BL/6NHsd mice were purchased from Harlan Sprague-Dawley at 6–7 weeks of age and individually housed for the remainder of their lifespan. Upon arrival to our facility, mice were fed one of two life-long dietary regimens: normal diet (ND) and caloric restriction (CR). Mice on ND were provided acidic water ad libitum and fed 84 kcal/wk which is ~15% less than the average ad libitum diet. C57BL/6NHsd mice on this dietary regiment typically have an average lifespan of 30 mo. in our colony. Mice on CR were fed a diet that resulted in a 26% difference in caloric intake to the ND animals. The diet fed to CR mice was enriched in protein, vitamins and minerals such that CR and ND mice were fed nearly identical amounts of these components. Detailed information on diet composition and feeding for ND and CR mouse diets have previously been described [49]. Five replicate mice were used for each diet and age group for microarray analysis. Dissected gastrocnemius muscle from the mice were flash-frozen in liquid nitrogen and stored at -80°C until processed for microarray or Western analysis.

### Tissue preparation and oligonucleotide array hybridization

RNA was extracted from frozen mouse tissue, transcribed to cDNA which was then used as a template to produce biotin-labeled cRNA as previously described [50]. A total of 10 μg of fragmented cRNA was used for hybridization to each MOE430A array (Affymetrix, Santa Clara, CA). After hybridization, the arrays were washed and stained with signal amplification protocol using antibody. The arrays were scanned twice and the averaged images were used for further analysis.

### Data analysis

The complete set of gene expression data has been deposited in the Gene Expression Omnibus database [51] accession # [GSE6323]. Robust Multi-Array Analysis (RMA) was used to pre-process and normalize the raw Affymetrix GeneChip data [52]. RMA is available in Bioconductor [53], an open source and open development software project for the analysis and comprehension of genomic data based primarily on the R programming language [54]. RMA fits a log-scale linear model to derive a single, normalized, summary score of expression for each probe on each array. RMA has been shown to provide greater sensitivity and specificity in detection of differentially expressed genes than the Affymetrix analysis software MAS 5.0 [52,54]. Because RMA generally provides attenuated estimates of fold-change [55], the values reported in the expression ratios for the array data are likely minimal estimates. MAS5.0 was used to compute absent/present calls. Any gene considered absent across all 15 arrays was removed from the analysis. This left 10,892 probes (out of 22,690).

### Statistical analysis

To identify genes differentially expressed over time, we used an empirical Bayes hierarchical modeling approach called EBarrays [56,57], also implemented in Bioconductor [54]. To briefly illustrate EBarrays, first consider the simple case of two biological conditions. For a given gene g, there are then two underlying levels of expression, one in each condition. These are the quantities of interest that are approximated by the expression measurements. Of most interest is the relationship between these means. In the case of equivalent expression (EE), the two means denoted by μg1 and μg2 are equal; μg1 ≠ μg2 denotes differential expression (DE). We refer to these equality and inequality relationships as expression patterns.

EBarrays assigns a probability to each gene for each pattern. To do this, the probability distribution governing the data must be described. A Gamma-Gamma (GG) model is often used. For that model, we assume the underlying expression levels are approximately distributed as Inverse Gamma; and, for a given gene g in condition k (in our simple example, k = 1,2), measured intensities arise from a Gamma distribution with mean μgk. The Inverse Gamma distribution here describes variation in the true underlying intensity levels of different genes (some genes have low expression, others high) and the Gamma distribution describes within gene variability due to biological and technical error variations. Similarly, a log-Normal Normal (LNN) model can also be used, where the underlying expression levels are assumed Gaussian, and log intensities for gene g in condition k are considered as Normally distributed with mean prescribed by μgk. Since we never know which genes are in which patterns, the full data distribution is given by a mixture: there is some probability p that the data for a given gene arise from pattern DE and a (1-p) chance that the data are EE.

For both cases, model parameters are determined by fitting the model to the full set of array data (details are given in [57]). In this way, the approach utilizes information across genes and arrays to optimize model fit, and is thus more efficient than a number of methods that make gene inferences one gene at a time. Once model parameters are obtained, probabilities for each pattern are calculated for every gene. These probabilities are referred to as posterior probabilities, since they are calculated after observing a set of data. For a given gene and expression pattern, the posterior probability quantifies evidence that the gene is in that pattern. Posterior probability thresholds can be set to control the false discovery rate (FDR) at a desired level [58]. For example, defining DE genes to be those with a posterior probability of DE exceeding 0.95 controls the FDR at 0.05.

This approach, described for two conditions, extends naturally to multiple conditions. Whereas for two biological conditions there are only the EE and DE patterns to consider, for multiple conditions there are many patterns. Given the three conditions here (young, old, and old CR), 5 expression patterns are possible (μ1 = μ2 = μ3; μ1 ≠ μ2 = μ3; μ1 = μ2 ≠ μ3; μ1 = μ3≠μ2; μ1≠μ2≠μ3). Model fit proceeds as in the two-pattern case (the details of the multiple pattern case are also given in [57]). Posterior probabilities for each of the 5 patterns were calculated for 10,892 genes under both GG and LNN assumptions. The appropriateness of model assumptions was checked using diagnostic plots. In most cases, results were similar for both models and only results from the LNN model are reported.

### Real-time (RT)-PCR

Total RNA was extracted from frozen tissue using the TRIZOL reagent (Life Technologies, Grand Island, NY). Polyadenylate [poly(A)+] RNA (mRNA) was then purified from the total RNA with oligo-dT-linked oligotex resin (Qiagen, Valencia, CA). Isolated mRNA was reverse transcribed to cDNA and amplified using the TaqMan EZ RT-PCR Kit (Applied Biosystems, Foster City, CA) (5 ng mRNA per 25 μl RT-PCR reaction volume). All primers and fluorescent probes for assayed genes, with the exception of the custom-made *p21 *and *p16*, were purchased from Assays-on-Demand Gene Expression probes (Applied Biosystems, Foster City, CA). The gene symbol and Applied Biosystem assay number for all Assays-on-Demand primer/probe sets used for RT-PCR are as follows: *gadd45 *(Mm00432802_m1), *p53 *(Mm00441964_g1), *bbc3 *(Mm00519268_m1), *pmaip1 *(Mm00451763_m1), *tnfrsf10b *(Mm00457866_m1), *bok *(Mm00479422_m1), *tbp *(Mm00446973_m1), and *gapd *(Mm99999915_g1). The sequence for the primers and probes for *p21 *and *p16 *are as follows: for *p21*, forward 5'-GGCAGACCAGCCTGACAGAT-3', reverse 5'-TTCAGGGTTTTCTCTTGCAGAAG-3', and probe 5'-TCTATCACTCCAAGGCA-3'; for p16, forward 5'- CCCAACGCCCCGAACT-3', reverse 5'- GCAGAAGAGCTGCTACGTGAA-3', and probe 5'-TTCGGTCGTACCCCGATTCAGGTG-3'. The sequence for the RT-PCR probe and primer for *p16 *was provided to us by Norman Sharpless's research group. The expression of *TATA box binding protein *(*tbp*) and *glyceraldehyde-3-phosphate dehydrogenase *(*gapd*), which showed approximately the same expression levels as measured on the microarrays for all animals regardless of age or diet, was used to normalize all RT-PCR results. RT-PCR was performed and quantified using the ABI PRISM 7000 Sequence Detection System (Applied Biosystems, Foster City, CA).

### Western immunoblot

Tissues homogenate was prepared from frozen in modified RIPA buffer (Tris-HCL 50 mM pH 7.4, NP-40 1%, Na-deoxycholate 0.25%, NaCl 150 mM EDTA 1 mM) containing protease and phosphatase inhibitors (Sigma). One hundred micrograms of each tissue extract were separated on 12.5% SDS PAGE gel (BioRad) and transferred to PVDF membrane (Pierce). Membranes were blocked in 5% milk in Tris buffered saline 0.1% Tween 20 (TBST) for 1 hour. Membranes were incubated in primary antibody overnight at 4°C with agitation in either 5% milk or 5% BSA in TBST at the indicated dilutions (GADD45A Santa Cruz Biotechnology Inc. 1:500; p21/WAF1 Ab-6 Calbiochem 1:4000; Tubulin Sigma 1:2000). After 3 × 5 min washes, the membranes were incubated in HRP conjugated secondary antibody (Vector labs, 1:10,000 dilution) for 1 hour at room temperature. Membranes were washed as before, incubated in SuperSignal West Pico Chemiluminescent Substrate (Pierce) and exposed to film (Pierce). Band density was calculated using NIH Image J software.

## Authors' contributions

ME performed most of the experiments in the paper and wrote the first draft and contributed to all subsequent drafts of the manuscript. RA performed all of the Western work. CK and MY performed the statistical analysis on the microarray data. RW provided all of the mice used in the study and served as advisor for this project. TP was the overall director of the research, provided the funds for this study, and contributed to the writing and editing of this manuscript. All authors have read and approved of the final manuscript.

## Supplementary Material

Additional file 1 Transcripts with age effect in mouse gastrocnemius muscle. Table of transcripts, listed alphabetically, that increased or decreased significantly (PP > 0.90) with age in mouse gastrocnemius muscle. Click here for file

Additional file 2 Expression of p53-related genes in wild type and transgenic GPX4 and MnSOD mice. Relative expression ratio ± SE for mRNA levels, as determined by RT-PCR, for p21, p16, Gadd45a and p53 in (**a**) 5 month old *GPX+/+ *and *GPX+/- *and (**b**) 9 month old *SOD+/+ *and *SOD+/- *gastrocnemius muscle (n = 5 for each group). Only the expression of p53 was considered significantly different (Student's T-Test; *P < 0.05) between *GPX+/+ *and *GPX+/- *mice. Click here for file

Additional file 3 Expression of p53-mediated proapoptotic genes in wild type and transgenic GPX4 and MnSOD mice. Relative expression ratio ± SE for mRNA levels, as determined by RT-PCR, for Bbc3 (Puma), Pmaip1 (Noxa), Tnfrsf10b (Killer/Dr5) and Bok in (**a**) 5 month old *GPX+/+ *and *GPX+/- *and (**b**) 9 month old *SOD+/+ *and *SOD+/- *gastrocnemius muscle (n = 5 for each group). Only the expression of Tnfrsf10b was considered significantly different (Student's T-Test; *P < 0.05) between *GPX+/+ *and *GPX+/- *mice. Click here for file

## Figures and Tables

**Table 1:** Biological classes of probe sets with an age effect on transcription levels in young and old mouse muscle.

	**# transcripts with age effect**		**P-value**		
			
**Biological Process**	**↓Old**	**↑Old**	**↓Old**	**↑Old**	**Ave. % CR Prevention**
lipid metabolism (4.5%)	19	13	<0.0005	NS	101 ± 9
protein metabolism (16.3%)	55	61	NS	NS	88 ± 5
carbohydrate metabolism (2.7%)	12	7	<0.05	NS	91 ± 15
energy pathways (1.7%)	10	2	<<0.0005	NS	100 ± 20
stress response (5.2%)	13	24	NS	<0.05	90 ± 8
immune response (3.4%)	4	20	NS	<0.005	99 ± 10
apoptosis (3.7%)	5	21	NS	<<0.0005	62 ± 15
**cell cycle (5.2%)**	9	28	NS	<0.005	66 ± 8
cell adhesion (4.1%)	18	11	<0.05	NS	49 ± 14
cytoskeleton organization and biogenesis (3.9%)	10	18	NS	<0.005	102 ± 12

Given in the above table are some of the more predominate biological processes, as determined by Gene Ontology [59], represented in the probe sets considered differentially expressed (PP > .90) in young and old mouse skeletal muscle. The number in parentheses is the percent each biological class represents out of all probe sets shown to have an age effect (712 total probe sets). Because genes can have multiple functions, many probe sets are associated with more than one biological process. Listed in the two middle columns on the left are the number of transcripts whose expression either decreased (↓ Old) or increased (↑ Old) in older mouse muscle. The middle columns on the right give the probability of obtaining the given transcript numbers for each biological class by chance. The last column gives the average CR prevention ± SE for all of the probe sets associated with these biological classes. The % CR prevention was calculated for each gene in the following manner: 100 × (old expression levels - CR expression levels)/(old expression levels - young expression levels). NS = not significant.

**Table 2:** Age-related effects on expression of p53-related genes in young and old C57BL/6 mouse gatrocnemius tissue.

**Gene**	**Expression Ratio**	**% CR Prevention**	**Posterior Probability**	**Representative Public ID**
*Genes That Encode Proteins That Bind P53*				
myogenic differentiation 1	1.52	101	0.9983	NM_010866
pleiomorphic adenoma gene-like 1	1.59	153	0.9987	AF147785
*Genes Whose Expression is Activated by P53 Activity*				
p21	1.92	67	1.0000	AK007630
GADD45a	1.79	44	1.0000	NM_007836
DNA-damage-inducible transcript 4	1.39	-2	0.9980	AK017926
dual specificity phosphatase 1	1.51	43	0.9992	NM_013642
IGF binding protein 3	1.28	106	0.9875	AV175389
keratin complex 1, acidic, gene 15	1.26	94	0.9890	NM_008469
mcl1	1.44	52	0.9894	BC003839
mcl1	1.45	27	0.9790	AV274748
mcl1	1.46	11	0.9539	AV318494
p53 apoptosis effector related to Pmp22	1.75	133	1.0000	NM_022032
sestrin 1	1.43	103	1.0000	BG076140
sestrin 1	1.44	60	0.9999	AV016566
pten	1.46	114	0.9798	AA214868
paternally expressed 3	1.52	99	0.9962	BM200248
retinoblastoma 1	1.50	63	1.0000	NM_009029
*Genes That Inhibit P53 Activity*				
B-cell leukemia/lymphoma 6	0.60	130	1.0000	U41465
cyclin G1	0.63	110	0.9980	BG065754
dual specificity phosphatase 10	0.48	46	1.0000	NM_022019
dual specificity phosphatase 10	0.66	49	0.9990	NM_022019
mdm2	0.63	90	0.9999	X58876

The above p53-related genes were selected from a list of 712 transcripts that were determined to be differentially expressed in young and old gastrocnemius mouse tissue using an empirical Bayes (EB) hierarchical modeling approach called EBarrays [56, 57] at posterior probability (PP) > .90. Genes listed more than once represent independent probes on the MOE430A array for the same gene identified in this statistical screen. Expression ratio = old (O) expression levels/young (Y) expression levels.

**Table 3:** Age-associated effects on the transcript levels of p53-related genes in various tissues of C57Bl6/J mice.

**Expression Ratio (%CR Prevention)**				
	**p21**	**p16**	**p53**	**Pmaip1**
**muscle**	2.2*(38%)	2.0*(65%)	1.4*(115%)	2.7**(79%)
**heart**	3.3*(104%)	2.6**(42%)	NSC	1.6*(104%)
**lung **	NSC	4.2*(62%)	NSC	1.7 (65%)
**liver**	2.6 (103%)	5.7**(23%)	1.5 (349%)	1.6 (116%)
**kidney**	1.6*(-37%)	11.2*(55%)	1.3*(94%)	1.4 (136%)
**brain**	NSC	6.6**(28%)	NSC	1.7**(34%)
**intestine**	1.5	40.6**	NSC	6.8*

Listed above are genes that are components of the p53 signaling pathway that show an age effect on expression in multiple mouse tissue-types. Expression ratio (O/Y) information is given for each gene if there was a significant difference (P < 0.05) in transcript levels, as determined by RT-PCR, between young and old or if this difference exceeded 1.2 fold and there was at least a 50% caloric restriction (CR) prevention. A total of 5 mice were used for each age group and diet (mRNA from CR mice were pooled together) for heart and muscle while 4 mice per age and diet were used for the remaining tissue types. No intestinal samples were available for old CR mice and therefore no values are given for this tissue for CR prevention. Statistical significance was determined using two-tailed Student's *t*-test, *P < 0.05; **P < 0.005. NSC = no significant change.
