# Supplementary material for: Gene expression profiling of aging reveals activation of a p53-mediated transcriptional program
Source: BMC Genomics. 2007 Mar 23;8:80. doi: 10.1186/1471-2164-8-80 (PMC1847444; doi:10.1186/1471-2164-8-80)
Supplement: Additional file 1 — Transcripts with age effect in mouse gastrocnemius muscle. Table of transcripts, listed alphabetically, that increased or decreased significantly (PP > 0.90) with age in mouse gastrocnemius muscle. [file 1471-2164-8-80-S1.pdf]

Transcripts with Age Effect in Mouse GN Muscle

Transcripts decreased with age (PP > 0.90) in male C57BL/6 GN muscle

Gene Name	Expression Ratio[1]	% CR Prevention[2]	Posterior Probability	Representative Public ID	Affymetrix ID
0610042I15Rik	0.57	90	1.0000	NM_019661	1460191_at
1010001P06Rik	0.72	-47	0.9750	BC011108	1451707_s_at
1110001E17Rik	0.61	109	0.9996	BC014728	1424178_at
1110006I15Rik	0.46	59	1.0000	AW494906	1416033_at
1110007C05Rik	0.63	115	1.0000	NM_025368	1449046_a_at
1110028A07Rik	0.67	99	0.9502	AB054000	1451488_at
1110028E10Rik	0.54	67	1.0000	BB478892	1438860_a_at
1500003O03Rik	0.54	125	1.0000	NM_019769	1450007_at
1810016I24Rik	0.72	167	0.9221	BC021466	1451744_a_at
1810044O22Rik	0.63	86	0.9935	NM_025558	1417767_at
1810044O22Rik	0.64	91	1.0000	NM_025558	1448844_at
1810060J02Rik	0.65	91	1.0000	AA067702	1418459_at
1810073N04Rik	0.72	64	0.9093	AK007977	1429979_a_at
2,3-bisphosphoglycerate mutase	0.53	83	1.0000	NM_007563	1448119_at
2,3-bisphosphoglycerate mutase	0.69	58	0.9414	NM_007563	1415864_at
2410003P15Rik	0.55	135	1.0000	NM_018888	1416436_a_at
2610529H08Rik	0.61	175	1.0000	NM_026202	1420523_at
2700083B06Rik	0.63	135	0.9995	NM_026531	1450506_a_at
2810405F18Rik	0.63	91	0.9935	AW701004	1429110_a_at
2810405K02Rik	0.65	78	0.9845	AI836168	1423266_at
2810409H07Rik	0.74	154	0.9956	AK019142	1430053_a_at
3110070M22Rik	0.45	91	1.0000	BG068672	1426607_at
3-hydroxybutyrate dehydrogenase	0.66	128	0.9321	BF322712	1426959_at
4430402O11Rik	0.57	87	1.0000	BI452475	1425344_at
4833439L19Rik	0.58	78	1.0000	NM_029241	1422017_s_at
5430411K16Rik	0.58	58	1.0000	BM250711	1434282_at
5730408C10Rik	0.68	81	0.9610	AK017523	1430527_a_at
6-phosphofructo-2-kinase/fructose-2,6-biphosphatase 3	0.49	75	1.0000	AV282911	1456676_a_at
6-phosphofructo-2-kinase/fructose-2,6-biphosphatase 3	0.71	37	0.9321	NM_133232	1416432_at
9430063L05Rik	0.68	81	0.9986	AI639670	1460426_at
A kinase (PRKA) anchor protein 1	0.56	111	1.0000	BG067335	1418279_a_at
A930009M04Rik	0.70	103	0.9759	BB497484	1426590_at
AA415817	0.66	157	0.9916	BB026304	1436372_a_at
acidic nuclear phosphoprotein 32A	0.67	265	0.9996	AF022957	1450407_a_at
actin related protein 2/3 complex, 4	0.66	140	0.9995	BG145444	1423588_at
actin, alpha, cardiac	0.29	79	1.0000	NM_009608	1415927_at
acyl-CoA acyltransferase 2	0.41	152	1.0000	BB718075	1455061_a_at
acyl-CoA synthetase 1	0.70	72	0.9481	BI413218	1422526_at
AI413331	0.67	16	0.9993	AV246911	1434479_at
AI663987	0.60	100	1.0000	BB764994	1448692_at
alanyl-tRNA synthetase	0.55	110	1.0000	BC026611	1423685_at

Gene Name	Expression Ratio[1]	% CR Prevention[2]	Posterior Probability	Representative Public ID	Affymetrix ID
amyloid beta precursor-like protein 2	0.44	104	1.0000	M97216	1421889_a_at
amyloid beta precursor-like protein 2	0.62	97	0.9991	AK013376	1432344_a_at
anaphase-promoting complex subunit 5	0.60	148	1.0000	AK003821	1453307_a_at
angiopoietin-like 2	0.54	117	1.0000	BG244279	1421002_at
angiotensin receptor-like 1	0.64	-138	1.0000	BB483357	1438651_a_at
annexin A6	0.47	149	1.0000	AK013026	1429246_a_at
annexin A6	0.67	133	0.9664	NM_013472	1415818_at
aortic preferentially expressed gene 1	0.49	127	1.0000	AF215896	1451886_at
aquaporin 4	0.62	101	1.0000	U48399	1425382_a_at
arginine-tRNA-protein transferase 1	0.66	107	0.9978	BE309332	1420651_at
Arl6ip2	0.73	25	0.9504	NM_019717	1416794_at
arsA arsenite transporter, homolog 1	0.54	131	1.0000	NM_019652	1418292_at
Asb15	0.71	56	0.9898	NM_080847	1460281_at
Asb8	0.64	75	0.9981	AF398969	1424225_at
ATP citrate lyase	0.43	169	1.0000	BI456232	1425326_at
ATP citrate lyase	0.50	166	1.0000	BI456232	1451666_at
ATP citrate lyase	0.61	196	1.0000	BB276877	1439459_x_at
ATPase, H+ transporting, v0a1	0.64	100	0.9994	U13836	1460650_at
ATPase, H+ transporting, v0b	0.70	140	0.9530	NM_033617	1416769_s_at
ATPase, Na+/K+ transporting, a2	0.46	155	1.0000	BB462665	1452308_a_at
ATP-binding cassette D2	0.63	142	1.0000	NM_011994	1419748_at
AU040950	0.68	114	0.9913	AF353717	1421815_at
barrier to autointegration factor 1	0.67	121	0.9834	NM_011793	1421081_a_at
barrier to autointegration factor 1	0.69	125	0.9329	NM_011793	1421083_x_at
basic leucine zipper and W2 domains 1	0.65	90	0.9997	AV144956	1450846_at
basigin	0.64	162	0.9987	AV035166	1456616_a_at
Baz1b	0.65	139	0.9998	BB253608	1420975_at
B-cell leukemia/lymphoma 6	0.60	130	1.0000	U41465	1450381_a_at
C330021A05Rik	0.65	70	0.9998	BB303415	1434946_at
C630016B22Rik	0.68	58	0.9536	BC026867	1451794_at
calcium channel, voltage-dep, g6	0.51	98	1.0000	AV091458	1425730_at
calcium/calmodulin-dependent protein kinase II alpha	0.45	93	1.0000	X14836	1452453_a_at
capping protein muscle Z-line, beta	0.46	111	1.0000	AK007209	1453960_a_at
capping protein muscle Z-line, beta	0.67	106	0.9947	NM_009798	1417259_a_at
carnitine palmitoyltransferase 1b	0.69	145	0.9630	AF017174	1418328_at
catenin beta	0.69	73	0.9357	BI134907	1430533_a_at
cathepsin B	0.67	136	0.9916	M14222	1448732_at
cathepsin Z	0.70	148	0.9768	NM_022325	1417870_x_at
cathepsin Z	0.73	218	0.9654	NM_022325	1417868_a_at
CCR4-NOT transcription complex 8	0.66	97	0.9948	NM_026949	1421135_a_at
cell division cycle 42 homolog	0.75	141	0.9968	BF143638	1449574_a_at
centromere autoantigen B	0.68	149	0.9970	BC006628	1426051_a_at
chondroadherin	0.57	-21	1.0000	NM_007689	1420569_at
citrate synthase	0.57	121	1.0000	AB056479	1422577_at
COX15 homolog	0.63	73	0.9997	BC011509	1460376_a_at

Gene Name	Expression Ratio[1]	% CR Prevention[2]	Posterior Probability	Representative Public ID	Affymetrix ID
Cpeb4	0.69	70	0.9997	NM_026252	1449931_at
cut-like 1 (Drosophila)	0.60	86	0.9999	BC014289	1424668_a_at
cyclin G1	0.64	110	0.9980	BG065754	1450016_at
cysteine and glycine-rich protein 1	0.62	118	1.0000	BF124540	1425810_a_at
cysteinyl-tRNA synthetase	0.67	190	0.9952	AI317241	1452394_at
cytidine and dCMP deaminase domain containing 1	0.75	71	0.9957	BB222482	1429186_a_at
D site albumin promoter bp	0.64	58	0.9992	BC018323	1418174_at
D site albumin promoter bp	0.68	-15	0.9893	BB550183	1438211_s_at
D0H4S114	0.48	28	1.0000	BB369191	1436736_x_at
D0H4S114	0.58	-12	1.0000	D45203	1450839_at
D11Ertd99e	0.63	123	0.9976	C77389	1419812_s_at
D11Wsu68e	0.62	75	1.0000	NM_026776	1421051_s_at
D19Ertd721e	0.71	168	0.9672	AU041099	1436342_a_at
D19Wsu162e	0.66	159	0.9972	BC026369	1451743_at
D1Ucla3	0.71	132	0.9357	BB123403	1434134_at
D4Wsu53e	0.56	89	0.9791	BE447520	1448538_a_at
D5Ertd33e	0.64	131	0.9997	AK014348	1417265_s_at
D8Ertd812e	0.57	143	1.0000	BG069220	1437314_a_at
dehydrogenase/reductase 4	0.73	163	0.9736	NM_030686	1419382_a_at
diaphanous homolog 1	0.75	175	0.9471	NM_007858	1421143_at
diaphorase 1 (NADH)	0.57	73	1.0000	AF332060	1425329_a_at
DnaJ homolog, A, member 3	0.65	134	0.9969	AK004575	1420629_a_at
DnaJ homolog, B, member 5	0.58	98	1.0000	AI664344	1421961_a_at
Down syndrome critical region 1	0.58	156	1.0000	AF282255	1416601_a_at
Down syndrome critical region 1-like 1	0.70	87	0.9847	NM_030598	1450243_a_at
dual specificity phosphatase 10	0.48	46	1.0000	NM_022019	1417164_at
dual specificity phosphatase 10	0.62	49	0.9990	NM_022019	1417163_at
dystonin	0.52	98	1.0000	NM_134448	1421276_a_at
dystrobrevin alpha	0.68	162	0.9584	AF143542	1419223_a_at
EH-domain containing 2	0.68	86	0.9498	BC027084	1424158_at
Eif2s3x	0.47	97	1.0000	NM_012010	1421895_at
Eif4a1	0.69	167	0.9996	BI656407	1430980_a_at
erythrocyte protein band 4.1-like 2	0.71	107	0.9228	BE951907	1433492_at
erythrocyte protein band 7.2	0.75	194	0.9916	AF093620	1419099_x_at
exostoses (multiple)-like 1	0.68	94	0.9901	BB826950	1423305_at
fatty acid synthase	0.38	114	1.0000	AF127033	1423828_at
F-box and WD-40 domain protein 1B	0.52	77	1.0000	AY038079	1425461_at
Fc receptor, IgG, alpha chain tr	0.70	117	0.9377	NM_010189	1416978_at
fibrillin 1	0.74	-10	0.9539	AF007248	1425896_a_at
fibroblast growth factor receptor 1	0.73	166	0.9947	M65053	1425911_a_at
fibromodulin	0.57	28	1.0000	NM_021355	1415939_at
fibromodulin	0.61	13	1.0000	BB235530	1437685_x_at
fibromodulin	0.64	-11	0.9999	BB504826	1437324_x_at
fibromodulin	0.65	14	0.9998	BB483571	1437718_x_at
fibromodulin	0.65	11	0.9997	BB532202	1456084_x_at

Gene Name	Expression Ratio[1]	% CR Prevention[2]	Posterior Probability	Representative Public ID	Affymetrix ID
fibromodulin	0.74	13	0.9824	AV290700	1438966_x_at
fibronectin 1	0.75	-49	0.9696	BC004724	1426642_at
frizzled-related protein	0.62	68	1.0000	U91905	1416658_at
fucosidase, alpha-L- 1, tissue	0.69	91	0.9676	BB225704	1437772_s_at
Gadd45b	0.72	68	0.9954	AK010420	1450971_at
ganglioside-induced diff-asso-protein 1	0.76	99	0.9613	AU017649	1423329_at
Gltscr2	0.68	83	0.9439	BC017637	1451121_a_at
glutamate oxaloacetate transaminase 2	0.63	159	0.9999	U82470	1417716_at
glycerol-3-phosphate acyltransferase	0.59	72	1.0000	BC019201	1425834_a_at
glycerol-3-phosphate dehydrogenase 1	0.44	103	1.0000	BC019391	1416204_at
glycogen synthase 3	0.47	124	1.0000	NM_030678	1450196_s_at
growth hormone receptor	0.75	132	0.9864	M33324	1451871_a_at
GTP binding protein 2	0.70	100	0.9431	NM_019581	1416691_at
H1 histone family, member 0	0.75	171	0.9265	NM_008197	1450522_a_at
H19 fetal liver mRNA	0.55	94	1.0000	NM_023123	1448194_a_at
Hbs1-like	0.58	95	1.0000	AK012856	1453604_a_at
heat shock 27kDa protein 8	0.68	143	0.9980	AF250139	1417014_at
heat shock protein 105	0.61	130	1.0000	BI499717	1423566_a_at
heat shock protein 2	0.61	139	0.9989	AK012780	1429888_a_at
heat shock protein family 7	0.40	140	1.0000	BG968304	1421289_at
hemoglobin alpha, adult chain 1	0.70	-12	0.9853	NM_008218	1417714_x_at
high density lipoprotein bp	0.60	102	1.0000	C77256	1449615_s_at
high density lipoprotein bp	0.62	86	1.0000	BG065877	1415988_at
high mobility group box tf 1	0.62	81	1.0000	AK006835	1432143_a_at
Hipk3	0.73	103	0.9527	NM_010434	1419191_at
Hivep2	0.73	-27	0.9871	NM_010437	1422018_at
Hnrpr	0.69	148	0.9881	BB822465	1452030_a_at
HRAS-like suppressor	0.60	116	1.0000	NM_013751	1422919_at
hypothetical LOC211623	0.62	132	1.0000	BB609699	1452590_a_at
inner membrane protein	0.56	134	1.0000	BB222675	1429533_at
integral membrane protein 2A	0.63	99	0.9982	BI966443	1451047_at
integrin beta 6	0.74	47	0.9520	AK019511	1432281_a_at
interleukin 6 signal transducer	0.59	86	1.0000	AA717838	1421239_at
interleukin 6 signal transducer	0.62	108	1.0000	AA717838	1460295_s_at
Janus kinase 1	0.57	106	1.0000	BQ032637	1433804_at
karyopherin (importin) beta 1	0.71	167	0.9275	AW544889	1434357_a_at
Kcnj11	0.58	108	1.0000	U73626	1450515_at
keratocan	0.70	46	0.9995	NM_008438	1418063_at
lectin, mannose-binding, 1	0.73	55	0.9623	BG071597	1428129_at
LIM domain binding 3	0.64	70	0.9948	AF114378	1451999_at
Lutheran blood group	0.72	119	0.9874	BC004826	1424791_a_at
MAD homolog 3 (Drosophila)	0.58	102	1.0000	BI150236	1450472_s_at
Map2k6	0.64	38	0.9999	BB261602	1426850_a_at
Mdm2	0.64	90	0.9999	X58876	1427718_a_at
methionyl aminopeptidase 1	0.70	93	0.9821	BG064851	1426837_at
microfibrillar associated protein 5	0.75	29	0.9758	NM_015776	1418454_at

Gene Name	Expression Ratio[1]	% CR Prevention[2]	Posterior Probability	Representative Public ID	Affymetrix ID
mitochondrial ribosomal protein S5	0.71	168	0.9133	AK018829	1453954_a_at
moesin	0.64	107	1.0000	NM_010833	1450379_at
multiple PDZ domain protein	0.66	130	0.9904	AK019164	1418663_at
myelin basic protein	0.63	94	0.9999	L07509	1451961_a_at
myelin protein zero	0.74	10	0.9826	AI385618	1423253_at
myosin, heavy polypeptide 6	0.72	297	0.9815	BB481540	1448827_s_at
myosin, light polypeptide kinase 2	0.61	100	0.9996	BC019408	1427556_at
neighbor of Brca1 gene 1	0.63	109	1.0000	AF227188	1451897_a_at
neural precursor cell expressed, 4	0.61	87	0.9998	NM_010890	1421955_a_at
nitrogen fixation gene 1	0.54	130	1.0000	AK003786	1431431_a_at
novel nuclear protein 1	0.54	148	1.0000	AV297071	1415957_a_at
nuclear receptor subfamily 1, D, 2	0.68	112	0.9990	NM_011584	1416959_at
nuclear receptor subfamily 3, C, 1	0.79	20	0.9911	NM_008173	1421866_at
nucleolin	0.49	170	1.0000	BF118393	1415773_at
open reading frame 28	0.68	109	0.9987	BC005604	1420542_at
opioid growth factor receptor	0.71	161	0.9385	AW476433	1422512_a_at
optic atrophy 1 homolog	0.63	53	0.9999	BC025160	1418768_at
ornithine decarboxylase antizyme 2	0.57	118	1.0000	AW214584	1426764_at
OTU domain, ubiquitin aldehyde binding 1	0.67	149	0.9932	NM_134150	1417705_at
peripheral myelin protein	0.75	17	0.9919	NM_008885	1417133_at
peroxisome biogenesis factor 19	0.59	107	1.0000	NM_023041	1448332_at
peroxisome biogenesis factor 5	0.64	87	0.9979	NM_008995	1422063_a_at
phosphate cytidylyltransferase 1a	0.64	120	1.0000	BC018313	1424453_at
phosphatidylinositol transfer protein	0.72	46	0.9962	AA239637	1423283_at
phosphodiesterase 7A	0.79	153	0.9029	AY007702	1451839_a_at
phospholipid transfer protein	0.68	70	0.9954	NM_011125	1417963_at
phosphorylase kinase gamma	0.73	62	0.9769	J03293	1425164_a_at
platelet derived gf receptor, alpha	0.67	58	0.9190	AW537708	1421916_at
polymerase III polypeptide C	0.50	107	1.0000	BC026793	1451658_a_at
procollagen, type I, alpha 1	0.38	-25	1.0000	U08020	1423669_at
procollagen, type I, alpha 1	0.73	-27	0.9900	BI794771	1455494_at
procollagen, type I, alpha 2	0.38	-25	1.0000	BF227507	1423110_at
procollagen, type I, alpha 2	0.47	-23	1.0000	BF227507	1450857_a_at
procollagen, type III, alpha 1	0.49	-37	1.0000	AW550625	1427883_a_at
procollagen, type III, alpha 1	0.69	-47	0.9988	AW550625	1427884_at
procollagen, type V, alpha 1	0.75	-39	0.9904	AW744319	1416740_at
procollagen, type VI, alpha 1	0.71	-1	0.9661	NM_009933	1448590_at
procollagen, type VI, alpha 2	0.73	8	0.9346	BI455189	1452250_a_at
procollagen, type VI, alpha 3	0.78	-80	0.9034	AF064749	1424131_at
procollagen-proline, 2-oxoglutarate 4-dioxygenase, alpha 1 polypeptide	0.65	110	0.9999	AI314028	1426519_at
prohibitin	0.63	111	0.9992	AK010619	1417053_at
proteasome 26S subunit, 7	0.70	175	0.9959	BB034143	1451056_at
protein kinase, type I, alpha	0.66	95	0.9967	BC005697	1425550_a_at
proteoglycan 4	0.62	37	1.0000	NM_021400	1449824_at

Gene Name	Expression Ratio[1]	% CR Prevention[2]	Posterior Probability	Representative Public ID	Affymetrix ID
pyruvate carboxylase	0.65	143	0.9975	L09192	AFFX-PyruCarb Mur/L09192_MB_at
pyruvate dehydrogenase E1 alpha 1	0.49	134	1.0000	NM_008810	1449137_at
RAD23a homolog	0.65	141	0.9992	AK009733	1453623_a_at
ras homolog gene family, member C	0.70	132	0.9276	NM_007484	1448605_at
regulator of G-protein signaling 5	0.64	148	1.0000	BF585144	1420941_at
regulator of G-protein signaling 5	0.73	223	0.9979	BF585144	1420942_s_at
regulator of G-protein signaling 5	0.74	53	0.9565	BF585144	1420940_x_at
restin	0.71	93	0.9381	NM_019765	1450351_a_at
ribonucleotide reductase M1	0.71	104	0.9558	BB758819	1448127_at
ribosomal protein L3-like	0.64	114	0.9842	NM_025425	1449398_at
ribosomal protein S6 kinase, 2	0.65	83	0.9922	BB737182	1417543_at
ring finger protein 121	0.64	137	0.9981	BI871826	1426503_a_at
ring finger protein 14	0.46	96	1.0000	AK010162	1431030_a_at
RNA binding motif protein 6	0.69	98	0.9844	BB706030	1417213_a_at
S100 calcium binding protein A1	0.57	180	1.0000	BC005590	1417421_at
S100 calcium binding protein A1	0.62	222	0.9999	AI266795	1419814_s_at
SAR1a gene homolog 1	0.54	110	1.0000	BC005549	1423720_a_at
Sdbcag84	0.73	150	0.9125	BB556862	1423548_s_at
secretory leukocyte protease inhibitor	0.74	3	0.9959	NM_011414	1448377_at
Sel1,1 homolog	0.68	54	0.9915	AK005023	1430692_a_at
selenoprotein W, muscle 1	0.52	144	1.0000	NM_009156	1416521_at
Sema3c	0.66	27	0.9925	NM_013657	1420696_at
serine proteinase inhibitor, F, 1	0.67	-10	0.9999	NM_011340	1416168_at
serine proteinase inhibitor, H, 1	0.66	26	0.9997	BI220012	1450843_a_at
SET and MYND domain containing 1	0.54	129	1.0000	NM_009762	1421329_a_at
SH3-domain kinase binding protein 1	0.64	104	0.9998	AK007283	1431592_a_at
signal recognition particle receptor, B	0.59	120	1.0000	NM_009275	1450089_a_at
signal transducer and activator of transcription 5B	0.61	109	0.9999	BC024319	1422102_a_at
small nuclear ribonucleoprotein D3	0.70	72	0.9731	NM_026095	1422884_at
Smoc2	0.63	160	1.0000	AK006809	1431362_a_at
SNRPN upstream reading frame	0.39	84	1.0000	NM_033174	1421063_s_at
sodium channel, voltage-gated, 4a	0.51	137	1.0000	NM_133199	1450557_at
solute carrier family 16, member 3	0.66	70	0.9996	NM_030696	1449005_at
solute carrier family 2, member 4	0.54	119	1.0000	AB008453	1415959_at
Sparc	0.65	-40	0.9996	NM_009242	1416589_at
Sparc	0.72	-54	0.9790	NM_009242	1448392_at
speckle-type POZ protein	0.44	112	1.0000	BB815380	1416525_at
spermine oxidase	0.60	-44	1.0000	BC004831	1424268_at
SRY-box containing gene 6	0.54	76	1.0000	D61689	1426018_a_at
stearoyl-Coenzyme A desaturase 1	0.28	111	1.0000	NM_009127	1415965_at
stearoyl-Coenzyme A desaturase 1	0.51	114	0.9989	NM_009127	1415964_at
stearoyl-Coenzyme A desaturase 2	0.48	47	1.0000	BG060909	1415824_at
stearoyl-Coenzyme A desaturase 2	0.58	40	1.0000	BG060909	1415823_at
stearoyl-Coenzyme A desaturase 2	0.68	-9	0.9952	BG060909	1415822_at

Gene Name	Expression Ratio[1]	% CR Prevention[2]	Posterior Probability	Representative Public ID	Affymetrix ID
stress-induced phosphoprotein 1	0.62	130	0.9989	NM_016737	1415909_at
suppressor of K+ transport defect 3	0.68	113	0.9893	AK006072	1454168_a_at
synaptopodin 2	0.55	103	1.0000	NM_080451	1450828_at
T-box 14	0.61	101	1.0000	NM_011534	1422195_s_at
tenomodulin	0.66	36	0.9999	AF291655	1417979_at
tetratricopeptide repeat domain 11	0.66	161	0.9627	NM_025562	1416764_at
thioredoxin interacting protein	0.55	76	1.0000	AF173681	1415997_at
thymoma viral proto-oncogene 2	0.71	111	0.9689	NM_007434	1421324_a_at
thyroid hormone responsive SPOT14 homolog	0.62	161	0.9996	NM_009381	1422973_a_at
thyrotroph embryonic factor	0.66	65	0.9995	NM_017376	1450184_s_at
tissue inhibitor of metalloproteinase 3	0.71	124	0.9277	BI111620	1449334_at
titin-cap	0.60	124	1.0000	AK010167	1423145_a_at
toll interacting protein	0.59	92	1.0000	BB400304	1423048_a_at
transferrin receptor	0.51	147	1.0000	BB810450	1422966_a_at
transferrin receptor	0.53	106	1.0000	AK011596	1452661_at
transferrin receptor	0.62	134	1.0000	X57349	AFFX-TransRec Mur/X57349_3_at
transferrin receptor	0.63	86	1.0000	BB810450	1422967_a_at
transferrin receptor	0.71	102	0.9919	X57349	AFFX-TransRec Mur/X57349_5_at
trans-golgi network protein	0.72	136	0.9198	AI314055	1423308_at
transketolase	0.64	180	0.9998	AI314476	1451015_at
transketolase	0.71	236	0.9944	AV103412	1439443_x_at
transmembrane 4 superfamily 2	0.40	130	1.0000	AF052492	1417502_at
tripartite motif-containing 35	0.58	114	1.0000	AB060155	1425621_at
tropomyosin 1, alpha	0.76	101	0.9411	BM232388	1456623_at
tropomyosin 2, beta	0.62	188	0.9998	AK003186	1449577_x_at
troponin C, cardiac/slow skeletal	0.71	286	0.9924	NM_009393	1418370_at
tumor differentially expressed 1	0.65	100	0.9979	BM239368	1456080_a_at
tumor differentially expressed 2	0.56	121	1.0000	BM239422	1437513_a_at
tumor rejection antigen gp96	0.58	172	1.0000	BE995678	1438040_a_at
ubiquitin-conjugating enzyme E2G 2	0.63	115	1.0000	AF296657	1417033_at
ubiquitin-like 4	0.69	116	0.9481	BI650739	1424538_at
uncoupling protein 3, mitochondrial	0.63	144	1.0000	AF053352	1420658_at
unknown	0.57	57	1.0000	BC021831	1427820_at
unknown	0.65	122	1.0000	BF580235	1427798_x_at
unknown	0.66	100	0.9985	BF580235	1427797_s_at
vacuolar protein sorting 41 (yeast)	0.56	66	1.0000	BM240052	1437901_a_at
vesicle-associated membrane protein 3	0.70	64	0.9995	NM_009498	1421102_a_at
vinculin	0.50	77	1.0000	NM_009502	1416157_at
WAP four-disulfide core domain 1	0.70	118	0.9117	AK018575	1431335_a_at
WD repeat domain 1	0.60	148	1.0000	AK004644	1423054_at
WD repeat domain 10	0.71	87	0.9843	AV319292	1427239_at
Ywhag	0.39	78	1.0000	NM_018871	1420816_at
Zfp106	0.46	77	1.0000	BI452653	1425331_at

Gene Name	Expression Ratio[1]	% CR Prevention[2]	Posterior Probability	Representative Public ID	Affymetrix ID
Zfp106	0.52	68	1.0000	BC025424	1425097_a_at
Zfp313	0.59	115	1.0000	NM_030743	1422959_s_at
Zmynd11	0.72	119	0.9383	BB832996	1426532_at

[1] Expression Ratio = old (O) expression levels/ young (Y) expression levels
[2] % Calorie restriction (CR) prevention = 100 X (O levels – CR levels)/ (O levels –Y levels)

Transcripts increased with age (PP > 0.90) in male C57BL/6 GN muscle

Gene Name	Expression Ratio[1]	% CR Prevention[2]	Posterior Probability	Representative Public ID	Affymetrix ID
0610011I04Rik	1.37	70	0.9979	BC006049	1425603_at
1100001G20Rik	1.31	136	0.9943	AV006463	1434484_at
1110005A23Rik	1.41	-51	0.9564	AV084012	1439438_a_at
1110007F12Rik	1.39	202	0.9993	BC020080	1424354_at
1110014F24Rik	1.82	153	1.0000	BI452905	1428781_at
1110021N07Rik	1.52	75	0.9967	BB417749	1437723_s_at
1110033F04Rik	1.49	115	0.9997	NM_026807	1421494_at
1110059H15Rik	1.51	42	0.9869	BB667784	1460455_at
1190002H23Rik	1.40	45	0.9779	NM_025427	1418003_at
1190002H23Rik	1.49	31	0.9981	BB408123	1438511_a_at
1300002F13Rik	1.67	61	1.0000	NM_133753	1416129_at
1600010O03Rik	1.47	33	0.9294	BI412951	1426407_at
1700024K14Rik	1.45	54	0.9813	BM217861	1435504_at
1810009M01Rik	1.37	108	0.9988	NM_023056	1418004_a_at
1810012N18Rik	1.56	80	0.9995	AV234245	1427131_s_at
1810015C04Rik	1.63	53	1.0000	BC019494	1424683_at
1810057C19Rik	1.64	121	1.0000	NM_026433	1449533_at
1-acylglycerol-3-phosphate O-acyltransferase 3	1.39	13	0.9628	NM_053014	1450504_a_at
2300002G24Rik	1.50	130	1.0000	AK009010	1453092_at
2310046G15Rik	1.31	92	0.9582	BB378796	1437671_x_at
2310075C12Rik	1.42	66	0.9976	NM_133739	1417222_a_at
2410026K10Rik	1.25	-37	0.9375	AK002762	1430514_a_at
2410026K10Rik	1.30	51	0.9677	AK004342	1428850_x_at
2410026K10Rik	1.36	25	0.9526	AK002762	1453556_x_at
2610103J23Rik	1.56	54	1.0000	AV083741	1434882_at
2610200G18Rik	1.46	106	0.9797	AK009389	1419651_at
3010033P07Rik	1.42	3	0.9904	AA762498	1434624_x_at
3110013H01Rik	1.42	40	0.9478	AK014046	1428706_at
3930401E15Rik	1.69	72	1.0000	AW545765	1419917_s_at
4732486I23Rik	1.41	70	0.9858	BC021511	1427151_at
4833420I20Rik	1.47	28	0.9825	AV217426	1456664_x_at
4930432O21Rik	1.45	123	0.9704	BM118654	1434625_at
4930586I02Rik	1.56	127	0.9983	AK006346	1417575_at
4933415F23Rik	1.40	32	0.9951	NM_025746	1420689_at
5730453H04Rik	1.75	176	1.0000	AV297961	1435493_at
5730453H04Rik	2.65	114	1.0000	AV297961	1435494_s_at
5830411E10Rik	1.40	91	0.9602	AV313559	1452203_at
9030221M09Rik	1.35	78	0.9894	AA673371	1434975_x_at
9030221M09Rik	1.40	57	0.9998	BB504983	1436944_x_at
9430059P22Rik	1.55	118	0.9998	BC024118	1423851_a_at
9430059P22Rik	1.74	137	1.0000	BC024118	1423852_at
9830124H08Rik	1.37	57	0.9814	BC023007	1425577_at
9930116P15Rik	1.47	77	0.9111	BG071611	1460711_at
A230103N10Rik	1.41	22	0.9332	BM940481	1426682_at

Gene Name	Expression Ratio[1]	% CR Prevention[2]	Posterior Probability	Representative Public ID	Affymetrix ID
AA408985	1.47	169	0.9021	AV024539	1433887_at
AB023956	1.53	149	1.0000	BB770932	1449070_x_at
Acdc	1.44	181	0.9997	NM_009605	1422651_at
actin-binding LIM protein 1	1.50	55	1.0000	BG065289	1454708_at
activating transcription factor 1	1.47	71	0.9682	NM_007497	1417296_at
activin A receptor, type 1	1.42	124	0.9552	NM_007394	1448460_at
activin receptor IIA	1.69	74	0.9999	BG066107	1437382_at
acyl-CoA synthetase 6	1.48	19	0.9910	BC022959	1451257_at
adipsin	1.51	134	0.9998	NM_013459	1417867_at
ADP-ribosylation factor 6	1.44	80	0.9776	BI248938	1418823_at
ADP-ribosylation factor 6	2.07	66	1.0000	BI248938	1418822_a_at
AI597080	1.52	97	1.0000	AI597080	1434425_at
AI642036	1.50	89	0.9974	AW537616	1452152_at
AI788669	1.36	121	0.9914	AA958965	1434312_at
aldehyde dehydrogenase family 1 A7	1.73	43	1.0000	NM_013467	1416468_at
amino-terminal enhancer of split	1.39	-43	0.9056	NM_010347	1420619_a_at
amylase 1, salivary	2.87	20	1.0000	NM_007446	1417765_a_at
angiomotin	1.55	4	1.0000	BG067039	1454890_at
ankyrin repeat domain 1	1.95	53	1.0000	AK009959	1420992_at
ankyrin repeat domain 1	1.28	-56	0.9974	AK009959	1420991_at
annexin A1	1.46	104	0.9959	NM_010730	1448213_at
annexin A8	1.31	147	0.9985	NM_013473	1417732_at
apolipoprotein D	1.80	57	1.0000	NM_007470	1416371_at
apolipoprotein E	1.19	52	0.9518	AK019319	1432466_a_at
ATPase, H+ transporting, ap2	1.41	36	0.9802	AV121721	1437688_x_at
ATPase, H+ transporting, v1av	1.34	62	0.9605	NM_007508	1422508_at
ATP-binding cassette A1	1.54	83	0.9903	BB144704	1421840_at
AU014947	1.60	76	0.9998	BM248774	1450897_at
AU022875	1.66	89	0.9998	BQ030867	1434513_at
AW111922	1.41	174	0.9965	BM239828	1419043_a_at
AW228700	1.40	132	0.9635	AF265663	1426979_at
AW548124	1.29	145	0.9707	BB323985	1454838_s_at
axotrophin	1.57	119	0.9978	NM_020575	1420609_at
B lymphoma Mo-MLV insertion region 1	1.56	87	0.9999	M64279	1417493_at
B lymphoma Mo-MLV insertion region 1	1.56	69	0.9990	M64279	1448733_at
B430320C24Rik	1.76	148	1.0000	BG066982	1435184_at
BC003331	1.71	81	1.0000	BI660899	1434471_at
BC010304	1.40	87	0.9099	AA673192	1433572_a_at
BC013529	1.47	34	0.9162	BC013529	1424025_at
BC013667	1.38	105	0.9909	BE987427	1424349_a_at
B-cell translocation gene 1	1.49	94	0.9894	AW322026	1437455_a_at
B-cell translocation gene 1	1.49	110	0.9995	L16846	1426083_a_at
beta-amyloid binding protein precursor	1.43	67	0.9060	AF353993	1426254_at
C/EBP alpha	1.37	173	0.9957	BC011118	1418982_at

Gene Name	Expression Ratio[1]	% CR Prevention[2]	Posterior Probability	Representative Public ID	Affymetrix ID
C/EBP, beta	1.82	68	1.0000	NM_009883	1418901_at
C/EBP, beta	2.10	57	1.0000	AB012278	1427844_a_at
C/EBP, delta	1.92	73	1.0000	BB831146	1423233_at
C030004A17Rik	1.37	2	0.9816	AK012261	1454236_a_at
C130006E22	1.54	12	0.9932	BQ175276	1455206_at
C1qtnf4	1.45	34	0.9939	NM_026161	1417050_at
C330005L02Rik	1.54	27	0.9997	BB503267	1419657_a_at
C630002M10Rik	1.30	101	0.9920	NM_019631	1422587_at
C730024G01Rik	1.43	52	0.9991	BE653749	1423577_at
C78339	1.56	100	0.9976	BG075168	1433475_a_at
C78339	1.57	55	0.9964	BG075168	1433476_at
cadherin 1	1.43	105	0.9999	NM_009864	1448261_at
calmodulin 1	1.47	52	0.9343	AV015462	1455571_x_at
calmodulin 1	1.53	87	0.9889	AU079514	1417365_a_at
calmodulin 1	1.60	46	0.9879	BB058862	1454611_a_at
calmodulin 2	1.49	116	0.9963	NM_007589	1422414_a_at
calmodulin 4	1.59	149	1.0000	NM_020036	1450633_at
carboxypeptidase D	1.62	111	0.9997	AW550842	1455009_at
catechol-O-methyltransferase	1.53	91	0.9509	NM_007744	1418701_at
cathepsin S	1.68	101	1.0000	NM_021281	1448591_at
CD163 antigen	1.38	62	0.9020	NM_053094	1419144_at
CD36 antigen	1.57	-2	0.9999	BB534670	1450884_at
CD36 antigen	1.72	1	1.0000	BB534670	1423166_at
CD36 antigen	1.73	25	0.9995	BB534670	1450883_a_at
CDC42 effector protein 3	1.41	134	0.9819	BB012489	1450700_at
chaperonin subunit 4 (delta)	1.52	58	0.9980	AV103196	1438560_x_at
chemokine (C-C motif) ligand 6	1.40	124	0.9991	BC002073	1417266_at
chemokine (C-C motif) ligand 8	1.63	72	1.0000	NM_021443	1419684_at
chemokine (C-X-C motif) ligand 13	1.29	50	0.9978	AF030636	1417851_at
chloride channel calcium activated 1	1.49	104	0.9999	AF108501	1460259_s_at
chloride channel calcium activated 1	1.64	120	1.0000	AF108501	1419463_at
chloride intracellular channel 4	1.95	35	1.0000	BB814844	1438606_a_at
cholinergic receptor, na1	1.53	-2	0.9999	NM_007389	1418852_at
cholinergic receptor, nb1	1.47	14	0.9996	M14537	1420682_at
Clock	1.38	16	0.9268	BB203106	1418659_at
coagulation factor XIII, alpha subunit	1.42	82	0.9280	NM_028784	1448929_at
cold shock domain protein A	1.59	17	0.9990	AV216648	1451012_a_at
colony stimulating factor 2rb2	1.66	95	1.0000	NM_007781	1449360_at
complement component 3	1.43	75	1.0000	K02782	1423954_at
complement component 4	1.31	110	0.9846	NM_009780	1418021_at
copine II	1.77	26	1.0000	BC023348	1424831_at
Cpeb2	1.54	29	0.9999	AV231491	1434272_at
Cpeb4	1.45	34	0.9578	NM_026252	1420618_at
CREBBP/EP300 inhibitory protein 1	1.44	31	0.9879	BC010712	1448406_at
CREBBP/EP300 inhibitory protein 1	1.47	73	0.9984	BC010712	1416614_at
CUG triplet repeat,RNA bp 2	1.43	54	0.9895	BB667096	1450069_a_at

Gene Name	Expression Ratio[1]	% CR Prevention[2]	Posterior Probability	Representative Public ID	Affymetrix ID
CUG triplet repeat,RNA bp 2	1.68	113	1.0000	BB644164	1451154_a_at
cyclin I	1.91	31	1.0000	NM_017367	1448334_a_at
D11Ertd730e	1.48	65	0.9799	BC025870	1451727_at
D130043N08Rik	1.41	183	0.9120	NM_007706	1418507_s_at
D230016N13Rik	1.47	51	1.0000	AI853240	1454842_a_at
D5Ertd363e	1.45	39	0.9552	AK012326	1452660_s_at
D5Ertd593e	1.43	-18	0.9986	BB667844	1434442_at
D7Wsu87e	1.50	121	0.9427	AJ250693	1415712_at
D7Wsu87e	1.71	100	1.0000	BG072903	1434705_at
D9Ertd392e	1.42	51	0.9797	BC006717	1424420_at
DEAD box polypeptide 3, Y-linked	1.50	41	0.9517	AA210261	1452077_at
DEAD box polypeptide 3, Y-linked	1.94	90	1.0000	AA210261	1426438_at
DEAD box polypeptide 5	1.38	-43	0.9816	AV025424	1454793_x_at
death-associated kinase 3	1.73	75	1.0000	AI642212	1423446_at
defensin beta 6	1.34	146	0.9908	NM_054074	1421807_at
dickkopf homolog 2	1.40	151	0.9561	NM_020265	1420512_at
DNA-damage-inducible transcript 4	1.46	-2	0.9980	AK017926	1428306_at
DnaJ homolog, B, member 4	1.46	39	0.9118	BC017161	1451177_at
dual specificity phosphatase 1	1.46	43	0.9992	NM_013642	1448830_at
dystonin	1.37	153	0.9592	NM_010081	1421117_at
E030026I10Rik	1.57	127	0.9997	BB832504	1448288_at
early growth response 1	1.32	124	0.9554	NM_007913	1417065_at
EGL nine homolog 3 (C. elegans)	1.36	-196	0.9692	BB284358	1418648_at
Eif3s1	1.46	-3	0.9772	BB379268	1426395_s_at
Eif3s10	1.51	54	0.9671	AW701127	1416659_at
Eif3s6	1.70	42	0.9999	BB032885	1439268_x_at
Eif5b	1.48	104	0.9796	BM236870	1434604_at
elongation factor RNA polymerase II 2	1.94	57	1.0000	NM_138953	1450744_at
Elovl4	1.47	129	1.0000	BB829575	1451308_at
Elovl4	1.84	119	1.0000	BB829575	1424306_at
embryonic ectoderm development	1.42	71	0.9786	U97675	1448653_at
Erbb2 interacting protein	1.69	6	1.0000	BC028256	1428011_a_at
fatty acid binding protein 5	1.33	-104	0.9995	BC002008	1416021_a_at
FBJ osteosarcoma oncogene	1.30	48	0.9889	AV026617	1423100_at
F-box only protein 14	1.39	63	0.9899	NM_133765	1417969_at
F-box only protein 22	1.65	13	0.9999	BB756840	1426593_a_at
Fc receptor, IgG, low affinity IIb	1.39	120	0.9928	BM224327	1435477_s_at
fetal Alzheimer antigen	1.50	78	0.9840	AA867746	1456615_a_at
fibroblast growth factor inducible 14	1.50	47	0.9836	BF123067	1423042_at
FK506 binding protein 5	1.58	69	1.0000	U16959	1416125_at
forkhead box O1	1.25	134	0.9967	AI462296	1416983_s_at
fragile X mental retardation syn 1	1.42	180	0.9601	BG172150	1423369_at
G0/G1 switch gene 2	1.56	-16	1.0000	NM_008059	1448700_at
Gadd45a	1.77	44	1.0000	NM_007836	1449519_at
gap junction alpha 1	1.35	93	0.9589	AV330726	1438650_x_at
gap junction alpha 1	1.45	77	0.9999	BB039269	1437992_x_at

Gene Name	Expression Ratio[1]	% CR Prevention[2]	Posterior Probability	Representative Public ID	Affymetrix ID
gap junction alpha 1	1.45	76	1.0000	BB142324	1438945_x_at
Gkap42	1.50	94	0.9920	NM_019832	1417594_at
glucan, branching enzyme 1	1.33	-37	0.9508	NM_028803	1420654_a_at
glucosamine (N-acetyl)-6-sulfatase	1.71	96	1.0000	BB543167	1433488_x_at
glutathione peroxidase 3	1.51	98	0.9190	NM_008161	1449106_at
GPI-anchored membrane protein 1	1.41	24	0.9912	BE981338	1448347_a_at
guanine nucleotide bp, alpha inhibiting 1	1.66	129	1.0000	BQ174580	1454959_s_at
heat shock protein 1, alpha	1.46	69	0.9945	C77287	1438902_a_at
heat shock protein 1A	1.27	27	0.9976	AW763765	1452388_at
heat shock protein 1B	1.44	56	0.9999	M12573	1452318_a_at
heat shock protein 1B	1.47	49	1.0000	M12573	1427126_at
heat shock protein 1B	1.74	47	1.0000	M12573	1427127_x_at
heat shock protein 4	1.40	95	0.9105	BE912771	1416146_at
histocompatibility 2, Aa	1.52	262	0.9963	BE688749	1435290_x_at
histocompatibility 2, T23	1.71	132	1.0000	NM_010398	1449556_at
histocompatibility 47	1.42	-17	0.9711	BB285733	1435735_x_at
Hnrpa1	1.49	54	0.9943	BI663320	1423531_a_at
Hnrpab	1.48	77	0.9717	NM_010448	1448144_at
Hnrpd	1.54	22	0.9999	BC011172	1425142_a_at
Hnrpdl	1.60	72	0.9990	BC021374	1424251_a_at
Hnrpr	1.30	25	0.9041	BB044174	1437526_x_at
homeo box D8	1.49	85	0.9973	AA265122	1431099_at
Ia-associated invariant chain	1.47	243	0.9303	BC003476	1425519_a_at
Igfbp3	1.26	106	0.9875	AV175389	1423062_at
Igfbp5	1.42	215	0.9070	BF225802	1452114_s_at
IK cytokine	1.43	62	0.9862	AV255179	1439271_x_at
immunoglobulin heavy chain 1a	1.51	68	1.0000	BC018365	1425385_a_at
immunoglobulin heavy chain 6	1.91	131	1.0000	AI326478	1427329_a_at
immunoglobulin heavy chain 6	3.03	123	1.0000	BB226392	1427351_s_at
immunoglobulin joining chain	2.16	108	1.0000	BC006026	1424305_at
immunoglobulin kappa chain V28	4.25	90	1.0000	BC013496	1427660_x_at
immunoglobulin kappa chain V28	4.55	91	1.0000	BI107286	1427455_x_at
immunoglobulin kappa chain V28	8.33	90	1.0000	AV057155	1452417_x_at
inhibitor of DNA binding 1	1.50	-11	0.9802	U43884	1425895_a_at
inhibitor of DNA binding 4	1.70	145	1.0000	BB121406	1423259_at
inhibitor of growth family 1-like	1.45	68	0.9893	NM_023503	1419111_at
integrin beta 5	1.46	141	0.9773	BB543979	1456195_x_at
interferon activated gene 203	1.42	96	0.9934	M74124	1426906_at
interferon activated gene 205	1.48	50	0.9972	AI481797	1452349_x_at
interferon-related dev regulator 1	1.41	30	0.9812	NM_013562	1416067_at
junctophilin 2	1.54	63	0.9977	NM_021566	1421453_at
keratin associated protein 16-8	1.51	121	1.0000	AF345298	1425237_at
keratin associated protein 6-1	1.30	93	1.0000	D86420	1451859_at
keratin complex 1, acidic, gene 10	2.18	147	1.0000	AK014360	1452166_a_at
keratin complex 1, acidic, gene 14	1.42	93	1.0000	BC011074	1460347_at

Gene Name	Expression Ratio[1]	% CR Prevention[2]	Posterior Probability	Representative Public ID	Affymetrix ID
keratin complex 1, acidic, gene 14	1.66	116	1.0000	BC011074	1423935_x_at
keratin complex 1, acidic, gene 15	1.36	94	0.9890	NM_008469	1422667_at
keratin complex 2, basic, gene 1	1.39	153	1.0000	NM_008473	1422481_at
keratin complex 2, basic, gene 17	1.78	166	1.0000	AV230775	1433923_at
keratin complex 2, basic, gene 5	1.59	151	1.0000	BC006780	1424096_at
keratin complex 2, basic, gene 6a	1.35	90	0.9993	NM_008476	1422784_at
keratinocyte diff. associated protein	1.61	168	1.0000	AV007306	1434227_at
kinesin family member 5B	1.51	81	0.9946	BI328541	1418429_at
Kruppel-like factor 2 (lung)	1.51	59	0.9998	NM_008452	1448890_at
Kruppel-like factor 4 (gut)	1.51	80	0.9950	BG069413	1417395_at
Kruppel-like factor 4 (gut)	1.97	50	1.0000	BG069413	1417394_at
large tumor suppressor 2	1.44	60	0.9343	BB134767	1439441_x_at
lectin, galactose binding, soluble 3	2.32	80	1.0000	X16834	1426808_at
leptin	1.37	105	0.9042	U18812	1422582_at
leucine-rich repeat-containing 2	1.45	103	0.9783	AJ428068	1427388_at
loricrin	1.31	194	1.0000	NM_008508	1448745_s_at
loricrin	1.36	117	0.9984	AI036317	1420183_at
LUC7-like 2	1.42	5	0.9234	BB475271	1436766_at
MAD homolog 1 (Drosophila)	1.44	100	0.9214	NM_008539	1448208_at
MAD homolog 4 (Drosophila)	1.50	108	0.9256	AK004804	1422487_at
MAD homolog 4 (Drosophila)	1.53	95	0.9739	AK004804	1422486_a_at
malonyl-CoA decarboxylase	1.46	100	0.9960	NM_019966	1449964_a_at
mannose receptor, C type 1	1.52	69	0.9988	NM_008625	1450430_at
Mapre2	1.48	51	0.9444	BC027056	1451989_a_at
Marcks	1.33	2	0.9241	BB454540	1456028_x_at
Marcks	1.40	14	0.9247	AW546141	1415972_at
Marcks	1.45	75	0.9800	BB100920	1456700_x_at
Marcks	1.54	171	0.9987	AW546141	1415971_at
matrin 3	1.46	43	0.9678	BM219545	1434888_a_at
matrin 3	1.48	9	0.9724	BF226671	1450874_at
matrin 3	1.70	18	1.0000	BB390675	1438368_a_at
metallothionein 1	1.47	52	1.0000	NM_013602	1422557_s_at
metallothionein 2	1.35	105	0.9926	AA796766	1428942_at
methyl-CpG binding domain protein 2	1.53	136	0.9993	AF072245	1425803_a_at
methyl-CpG binding domain protein 2	1.55	113	0.9969	NM_010773	1417165_at
Mll5	1.52	89	0.9959	BB529352	1434704_at
myeloid cell leukemia sequence 1	1.40	52	0.9894	BC003839	1416881_at
myeloid cell leukemia sequence 1	1.43	11	0.9539	AV318494	1437527_x_at
myeloid cell leukemia sequence 1	1.43	27	0.9790	AV274748	1456381_x_at
myogenic differentiation 1	1.58	101	0.9983	NM_010866	1418420_at
myosin heavy chain IX	1.32	23	0.9015	C80049	1420171_s_at
myosin, heavy polypeptide 3	1.28	36	0.9174	M74753	1427115_at
myosin, light polypeptide 4	1.68	57	1.0000	NM_010858	1422580_at
myotrophin	1.45	95	0.9495	NM_008098	1420474_at
myotrophin	1.47	96	0.9045	BB124537	1437457_a_at
nephronectin	1.43	122	0.9599	AA223007	1452106_at

Gene Name	Expression Ratio[1]	% CR Prevention[2]	Posterior Probability	Representative Public ID	Affymetrix ID
neuralized homolog	1.78	79	1.0000	AK010787	1453527_a_at
neuronatin	1.78	216	1.0000	AV218841	1423506_a_at
neuropilin	1.56	125	0.9926	AK011144	1448943_at
Nfkbia	1.53	89	0.9953	AI462015	1449731_s_at
nuclear receptor co-repressor 1	1.50	82	1.0000	U22016	1423201_at
nuclear receptor co-repressor 1	1.55	93	0.9998	U22016	1423200_at
nucleophosmin 1	1.61	60	0.9996	NM_008722	1415839_a_at
nucleosome assembly protein 1-like 1	1.44	79	0.9452	BG064031	1420477_at
oncostatin M receptor	1.73	112	1.0000	AB015978	1418674_at
ornithine decarboxylase, structural	1.58	145	0.9881	BB519474	1437711_x_at
p21 (cdkn1a)	1.88	67	1.0000	AK007630	1424638_at
p300/CBP-associated factor	1.50	60	0.9026	AV094898	1434037_s_at
pantothenate kinase 1	1.33	54	0.9821	BC023496	1418715_at
paternally expressed 3	1.55	99	0.9962	BM200248	1433924_at
PERP, TP53 apoptosis effector	1.83	133	1.0000	NM_022032	1416271_at
phosphatase and tensin homolog	1.42	114	0.9798	AA214868	1422553_at
phosphodiesterase 4B	1.52	52	0.9982	BM246564	1422473_at
phosphodiesterase 4B	1.95	78	1.0000	BM246564	1422474_at
phospholipase A2, group VII	1.48	98	0.9909	AK005158	1430700_a_at
phospholipase A2, group XIIA	1.40	43	0.9816	AY007382	1452026_a_at
plakophilin 4	1.35	57	0.9994	AV286396	1452209_at
plasmalemma vesicle associated protein	1.54	135	1.0000	NM_032398	1418090_at
pleiomorphic adenoma gene-like 1	1.65	153	0.9987	AF147785	1426208_x_at
polyhomeotic-like 2 (Drosophila)	1.51	96	0.9886	BB334118	1437239_x_at
premature ovarian failure 1B	1.64	106	1.0000	AF408412	1427492_at
procollagen, type IV, alpha 3 bp	1.40	86	0.9498	BC016197	1420384_at
procollagen, type XVII, alpha 1	1.64	138	1.0000	NM_007732	1418799_a_at
proline-rich nuclear receptor coactivator 1	1.42	132	0.9807	BI410130	1433668_at
proline-rich nuclear receptor coactivator 2	1.39	18	0.9854	NM_026383	1416187_s_at
proteasome subunit, alpha type 7	1.55	79	0.9948	BG297088	1423568_at
proteasome subunit, alpha type 7	1.66	60	0.9998	BG297088	1423567_a_at
protein phosphatase 1, regulatory 3C	1.70	6	1.0000	BQ176864	1433691_at
protein phosphatase 2a, catalytic, beta	1.43	94	0.9680	AI323685	1421823_a_at
protein phosphatase 4, regulatory 2	1.46	74	0.9886	BI735251	1433850_at
protein tyrosine phosphatase 4a1	1.61	62	0.9998	BC003761	1449322_at
protein tyrosine phosphatase 4a1	1.68	52	1.0000	BB043450	1438657_x_at
protein tyrosine phosphatase, n12	1.60	44	1.0000	X63440	1422045_a_at
protein tyrosine phosphatase, rF	1.51	136	1.0000	BF235516	1420842_at
protein tyrosine phosphatase-like b	1.41	171	0.9965	NM_023587	1449342_at
purine rich element binding protein A	1.42	142	0.9942	NM_008989	1420628_at
pyruvate dehydrogenase kinase 4	1.95	75	1.0000	NM_013743	1417273_at
quaking	1.28	-153	0.9282	NM_021881	1417073_a_at
RAB21	1.41	64	0.9252	BB767504	1437741_at
Rab40b	1.60	58	0.9976	AV364488	1436566_at

Gene Name	Expression Ratio[1]	% CR Prevention[2]	Posterior Probability	Representative Public ID	Affymetrix ID
RAB4A	1.47	137	0.9983	NM_009003	1418341_at
RAB6	1.55	85	0.9599	NM_024287	1448305_at
Rac1	1.49	139	0.9881	BC003828	1423734_at
RAN binding protein 2	1.43	64	0.9992	BM507707	1422621_at
ras homolog gene family, member B	1.33	13	0.9811	BC018275	1449110_at
ras homolog gene family, member J	1.45	73	0.9872	AF309564	1418892_at
ras homolog gene family, member U	1.29	13	0.9664	AF378088	1449028_at
ras homolog gene family, member U	1.40	45	0.9974	AF378088	1449027_at
RAS p21 protein activator 1	1.42	51	0.9281	AA124924	1426476_at
RASD family, member 2	1.70	5	1.0000	BC026377	1427343_at
RASD family, member 2	2.00	48	1.0000	BC026377	1427344_s_at
reduced expression 3	1.42	13	0.9914	NM_009052	1448595_a_at
retinoblastoma 1	1.50	63	1.0000	NM_009029	1417850_at
retinoblastoma binding protein 7	1.52	63	0.9018	AV310432	1456227_x_at
retinoic acid induced 14	1.43	98	0.9146	NM_030690	1417400_at
Rho GTPase activating protein 17	1.40	84	0.9731	AV351061	1426623_a_at
ribosomal protein L27a	1.76	47	1.0000	BG141806	1437729_at
ribosomal protein L5	1.39	46	0.9341	BM114165	1451077_at
ring finger protein 103	1.58	151	0.9995	NM_009543	1448434_at
RNA and export factor bp1	1.53	55	0.9999	NM_019484	1422993_s_at
Rock1	1.46	63	0.9670	BI662863	1423445_at
r-ras oncogene homolog 2	1.43	84	0.9755	NM_025846	1417398_at
S3-12	1.68	99	1.0000	NM_020568	1418595_at
salvador homolog 1	1.43	130	0.9815	NM_022028	1448204_at
sarcolipin	2.76	32	1.0000	AK008863	1420884_at
SCY1-like 1	1.37	102	0.9345	AU016501	1436804_s_at
sestrin 1	1.42	103	1.0000	BG076140	1454699_at
sestrin 1	1.44	60	0.9999	AV016566	1438931_s_at
SH3-binding domain glutamic rl	1.48	69	0.9966	BB248904	1436997_x_at
signal recognition particle 19	1.73	114	0.9997	W08076	1450891_at
sin3 associated polypeptide	1.58	102	0.9998	NM_021788	1417719_at
single-minded 2	1.47	67	0.9912	D63383	1419437_at
small proline-rich protein 1A	1.30	99	0.9983	NM_009264	1449133_at
solute carrier family 2, member 3	1.63	149	0.9956	BB414515	1437052_s_at
solute carrier family 25, member 28	1.54	83	0.9969	BC025908	1424776_a_at
solute carrier family 38, member 2	1.75	24	1.0000	BM248527	1426722_at
sorbin and SH3 domain containing 1	1.36	39	0.9966	BB737680	1436737_a_at
special AT-rich sequence bp 1	1.40	115	0.9635	AV172776	1416007_at
special AT-rich sequence bp 1	1.69	60	1.0000	AV172776	1416008_at
sperm specific antigen 2	1.47	50	0.9867	BB033597	1423613_at
S-phase kinase-associated protein 1A	1.54	-25	0.9141	AV347477	1423149_at
splicing factor proline/glutamine rich	1.44	-31	0.9940	AY034062	1423795_at
splicing factor, arginine/serine-rich 1	1.51	26	0.9922	BF147037	1428099_a_at
splicing factor, arginine/serine-rich 2	1.58	126	0.9993	AK011528	1415807_s_at
sterol carrier protein 2, liver	1.52	25	0.9939	C76618	1449686_s_at
synaptic nuclear envelope 1	1.43	83	0.9413	BI734306	1455493_at

Gene Name	Expression Ratio[1]	% CR Prevention[2]	Posterior Probability	Representative Public ID	Affymetrix ID
synaptotagmin binding, cytoplasmic RNA interacting protein	1.38	15	0.9334	BB469322	1426402_at
syndecan 1	1.33	150	0.9162	BB533095	1437279_x_at
syntaxin 6	1.47	123	0.9887	BB492711	1460004_x_at
TAF10 RNA polymerase II	1.49	53	0.9935	NM_020024	1448784_at
TCDD-inducible poly polymerase	1.42	79	0.9892	BB707122	1452160_at
telomerase binding protein, p23	1.48	7	0.9984	BC003708	1417998_at
TGFB inducible early growth response 1	1.47	8	1.0000	NM_013692	1416029_at
thioredoxin-like 5	1.38	45	0.9925	AV339214	1439184_s_at
thymoma viral proto-oncogene 1	1.43	-113	0.9999	M94335	1425711_a_at
tissue inhibitor of metalloproteinase 4	1.48	47	1.0000	BI788452	1423405_at
topoisomerase (DNA) I	1.50	42	0.9444	BG068053	1423474_at
transducer of ERBB2, 2	1.41	-29	0.9962	AV174616	1448666_s_at
transforming growth factor, beta 2	1.43	78	0.9727	BF144658	1450922_a_at
transglutaminase 2, C polypeptide	1.45	122	0.9993	BB041811	1455900_x_at
transglutaminase 2, C polypeptide	1.50	125	0.9765	BB550124	1437277_x_at
transmembrane 9 superfamily 2	1.62	58	0.9958	BB131843	1455875_x_at
tubulin cofactor a	1.46	-24	0.9905	BB559082	1437907_a_at
tumor rejection antigen gp96	1.42	-24	0.9504	NM_011631	1415889_a_at
ubiquilin 2	1.66	89	1.0000	AV171029	1450021_at
ubiquitin protein ligase E3A	1.39	60	0.9965	AK018443	1416680_at
ubiquitin specific protease 25	1.44	38	0.9231	NM_013918	1448939_at
ubiquitin-conjugating enzyme E2M	1.41	40	0.9071	BC021792	1424345_s_at
UDP-glucuronosyltransferase 1, 2	1.35	159	0.9619	D87867	1426260_a_at
unknown	1.22	103	0.9507	AV084904	1420249_s_at
unknown	1.42	39	0.9998	BB119177	1435137_s_at
unknown	1.44	80	0.9816	C80049	1420172_at
unknown	1.44	84	0.9999	BG862223	1455869_at
unknown	1.52	108	0.9991	BC004065	1452426_x_at
unknown	1.55	90	0.9983	BF453911	1436337_at
unknown	3.37	83	1.0000	BG966217	1452463_x_at
v-crk oncogene homolog (avian)	1.46	62	0.9691	BF020879	1416201_at
v-crk oncogene homolog (avian)	1.58	45	0.9987	BF020879	1448248_at
Von Willebrand factor homolog	1.41	176	0.9997	BB667216	1435386_at
Wnt4	1.72	81	0.9998	NM_009523	1450782_at
X-box binding protein 1	1.50	104	0.9854	AV051768	1437223_s_at
YY1 transcription factor	1.62	74	0.9999	BI665246	1422569_at
Zfp36l1	1.47	66	0.9989	M58566	1450644_at
Zfp622	1.49	122	0.9787	BB026724	1438000_x_at
Zmynd11	1.41	58	0.9592	BB561053	1436153_a_at
zuotin related factor 2	1.50	54	0.9916	BG067003	1448794_s_at

[1] Expression Ratio = old (O) expression levels/ young (Y) expression levels
[2] % Calorie restriction (CR) prevention = 100 X (O levels – CR levels)/ (O levels –Y levels)
