# Supplementary figures and images for: Gene expression profiling of aging reveals activation of a p53-mediated transcriptional program
Source: BMC Genomics. 2007 Mar 23;8:80. doi: 10.1186/1471-2164-8-80 (PMC1847444; doi:10.1186/1471-2164-8-80)

**A**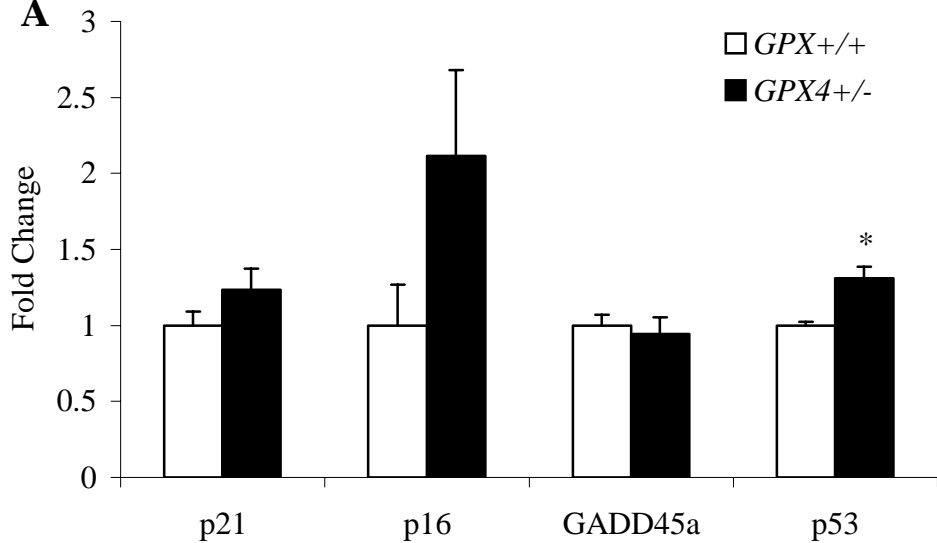**B**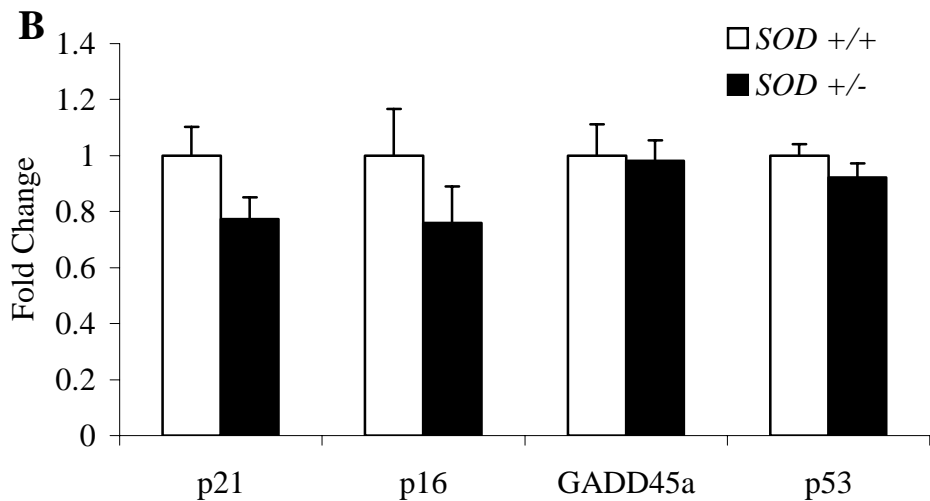

Supplement: Additional file 2 — Expression of p53-related genes in wild type and transgenic GPX4 and MnSOD mice. Relative expression ratio ± SE for mRNA levels, as determined by RT-PCR, for p21, p16, Gadd45a and p53 in (a) 5 month old GPX+/+ and GPX+/- and (b) 9 month old SOD+/+ and SOD+/- gastrocnemius muscle (n = 5 for each group). Only the expression of p53 was considered significantly different (Student's T-Test; *P < 0.05) between GPX+/+ and GPX+/- mice. [file 1471-2164-8-80-S2.pdf]

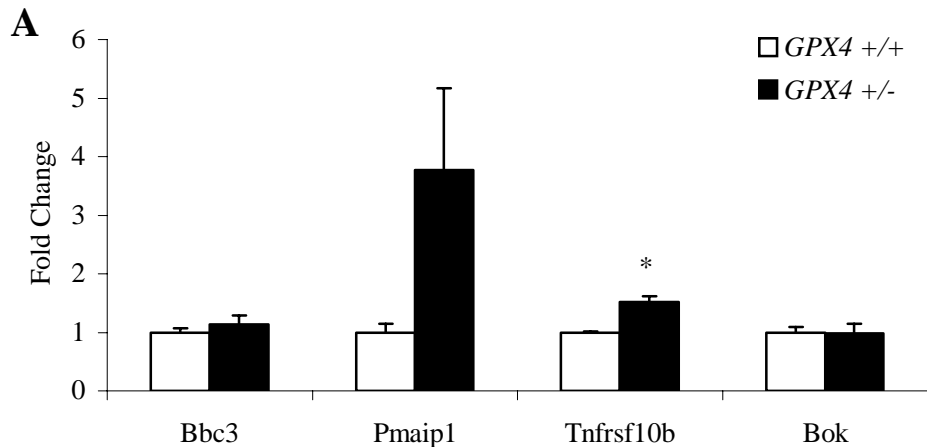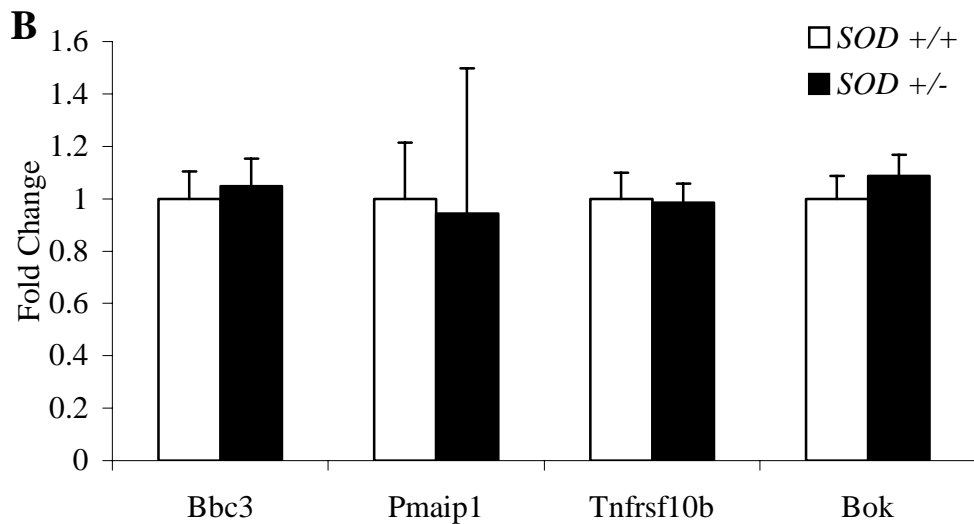

Supplement: Additional file 3 — Expression of p53-mediated proapoptotic genes in wild type and transgenic GPX4 and MnSOD mice. Relative expression ratio ± SE for mRNA levels, as determined by RT-PCR, for Bbc3 (Puma), Pmaip1 (Noxa), Tnfrsf10b (Killer/Dr5) and Bok in (a) 5 month old GPX+/+ and GPX+/- and (b) 9 month old SOD+/+ and SOD+/- gastrocnemius muscle (n = 5 for each group). Only the expression of Tnfrsf10b was considered significantly different (Student's T-Test; *P < 0.05) between GPX+/+ and GPX+/- mice. [file 1471-2164-8-80-S3.pdf]
